# How to use COVID-19 antiviral drugs in patients with chronic kidney disease

**DOI:** 10.3389/fphar.2023.1053814

**Published:** 2023-02-09

**Authors:** Ajinath Kale, Vishwadeep Shelke, Neha Dagar, Hans-Joachim Anders, Anil Bhanudas Gaikwad

**Affiliations:** ^1^ Laboratory of Molecular Pharmacology, Department of Pharmacy, Birla Institute of Technology and Science Pilani, Pilani Campus, Rajasthan, India; ^2^ Division of Nephrology, Department of Internal Medicine IV, Hospital of the Ludwig Maximilians University Munich, Munich, Germany.

**Keywords:** COVID-19, antivirals, chronic kidney disease, clinical trials, pharmacokinetics, pharmacodynamics, monoclonal antibodies

## Abstract

Antiviral drugs such as Remdesivir (Veklury), Nirmatrelvir with Ritonavir (Paxlovid), Azvudine, and Molnupiravir (Lagevrio) can reduce the risk for severe and fatal Coronavirus Disease (COVID)-19. Although chronic kidney disease is a highly prevalent risk factor for severe and fatal COVID-19, most clinical trials with these drugs excluded patients with impaired kidney function. Advanced CKD is associated with a state of secondary immunodeficiency (SIDKD), which increases the susceptibility to severe COVID-19, COVID-19 complications, and the risk of hospitalization and mortality among COVID-19 patients. The risk to develop COVID-19 related acute kidney injury is higher in patients with precedent CKD. Selecting appropriate therapies for COVID-19 patients with impaired kidney function is a challenge for healthcare professionals. Here, we discuss the pharmacokinetics and pharmacodynamics of COVID-19-related antiviral drugs with a focus on their potential use and dosing in COVID-19 patients with different stages of CKD. Additionally, we describe the adverse effects and precautions to be taken into account when using these antivirals in COVID-19 patients with CKD. Lastly, we also discuss about the use of monoclonal antibodies in COVID-19 patients with kidney disease and related complications.

## 1 Introduction

Coronavirus disease (COVID-19) is a transmissible disease caused by different variants of SARS-CoV-2 viruses: α, β, γ, δ, omicron, and its BA subtypes (Dhawan and PriyankaChoudhary, 2022). Delta and omicron have been declared as variants of concern (VOC) by the World Health Organization (WHO) and the Centers for Disease Control and Prevention (CDC). These variants are more perilous and contagious compared to previous variants (Duong et al., 2022). Figure 3 gives an overview of SARS-CoV-2’s life cycle. The WHO notified its outbreak on 30/1/2020 as a worldwide health emergency; however, on 11/3/2020, it was declared a pandemic due to the rapid spread and mortality due to infection (Capone et al., 2020). As per the WHO, to the date (24 September 2022) the number of COVID-19 cases reached 619 million wherein 6.539 million deaths are recorded. Moreover, the mortality rate is highest among comorbid and elderly patients. As per a meta-analysis of 46,248 infected patients, hypertension (17%), diabetes (8%), preceded by cardiovascular disorders (5%), and lung disease (2%) were reported as the most prominent comorbidities and deemed as the main risk factors for COVID-19 (Capone et al., 2020). However, reports from the years 2021–2022 revised these initial impressions and revealed that advanced chronic kidney disease (CKD) is an important independent risk factor for severe and fatal COVID-19 (Henry and Lippi, 2020; Ortiz, 2021). Advanced CKD is associated with a state of secondary immunodeficiency (SIDKD), which increases the susceptibility to severe COVID-19, COVID-19 complications, and the risk of hospitalization and mortality among COVID-19 patients (Henry and Lippi, 2020; Steiger et al., 2022). The risk to develop COVID-related acute kidney injury (AKI) is higher in patients with precedent CKD (Thakare et al., 2021). Selecting appropriate therapies for COVID-19 patients with impaired kidney function is a challenge for all healthcare professionals.

The foremost treatment options for COVID-19 include vaccination, anti-inflammatory agents, anticoagulants, monoclonal antibodies, and antiviral therapies alone or in combination (Drożdżal et al., 2021). Moreover, other classes of drugs are currently being tested for their suitability and efficacy in ongoing interventional clinical trials. Unfortunately, most of the clinical studies exclude COVID-19 patients with precedent CKD hence reliable pharmacokinetic and pharmacodynamics data is not easily accessible for the use of these drugs in COVID-19 patients with CKD (Major et al., 2020).

Antiviral drugs such as Remdesivir (Veklury), Nirmatrelvir with Ritonavir (Paxlovid), Azvudine, and Molnupiravir (Lagevrio) are recommended by international and national health agencies and more antivirals are in the pipeline for COVID-19 (www.cdc.gov, www.who.int, www.fda.gov). Atazanavir (NCT04468087), oseltamivir (NCT04973462), famciclovir (https://medsafe.govt.nz), darunavir (NCT04425382), sofosbuvir (NCT04460443), favipiravir (NCT04351295), ribavirin (NCT04494399), umifenovir (NCT04350684), and lopinavir/ritonavir (NCT0437693) have therapeutic potential and has already been approved/being tested against several viral diseases/infections. However, insufficient inadequate and contradictory information is available regarding the dosage, frequency, and safety of these drugs to use in CKD. In patients with CKD, these antivirals may produce more nephrotoxicity or more adverse effects compared to patients with normal kidney functions (Jonny et al., 2021). Hence, information about the safety, applicability, dosage, frequency, and efficacy of such drugs, and guidelines for their usage in CKD need to be established.

This review discusses the known and the unknown about the pharmacokinetics, pharmacology, rationale, and limitations/adverse effects of COVID-19 antiviral drugs. We reviewed the antiviral drug-related information from different databases and official websites such as ClinicalTrials.gov (https://www.ClinicalTrials.gov), U.S. Food & Drug Administration (https://www.fda.gov), WHO (https://covid19.who.int), CDC (https://www.cdc.gov), PubMed (https://pubmed.ncbi.nlm.nih.gov), IDSA (https://www.idsociety.org), Web of Science (https://www.webofscience.com/wos/woscc/basic-search), original Journal’s site (by whom/wherein paper is published), and ScienceDirect (https://www.sciencedirect.com). We reviewed clinical trials, clinical reports, official guidelines, and reviews between the period of 1993–2022. Only articles in English were considered. All articles meeting these criteria were analyzed for demographic data and clinical outcomes. Based on the pharmacological characteristics of each of these drugs, we make suggestions regarding the applicability, effectiveness, dosage, frequency, precautions, and limitations of these antiviral drugs for the treatment of COVID-19 patients with CKD (Table 1).

**TABLE 1:** Recommendations for dosing and precautions of antivirals in COVID-19 patients with different stages of CKD.

S.N.	Drug name	Trial stage	CKD stages	Recommended dose/adjustment of dose		Limitations/precautions/adverse effects
1	Remdesivir	Phase III/IV US-FDA issued a EUA	G1	100%		No dose adjustment is required when given by nasal route
			G2	100%		
			G3	100%		
			G4	Not recommended		Prothrombin time shall be monitored
			G5	Not recommended		Nausea
			G5D	Not recommended		ALT and AST elevations HSR
			Risk for nephrotoxicity			Higher doses by the i.v. route may cause nephrotoxicity
			Risk for accumulation and systemic toxicity			It may cause liver toxicity. Hence liver function should be monitored
2	Atazanavir (ATV)	Phase II/III	G1	100%		Booster dose after dialysis
			G2	100%		
			G3	100%		Warfarin, simvastatin, irinotecan, lovastatin, and phosphodiesterase inhibitors may show drug interactions with ATV.
			G4	100%		
			G5	100%		
			G5D	Dose adjustment required		
			Risk for nephrotoxicity			Chronic exposure to ATV can lead to granulomatous interstitial nephritis and glomerulosclerosis
			Risk for accumulation and systemic toxicity			ATV can cause allergic reactions that manifest as rash, toxic skin eruptions, erythema multiforme, or Stevens-Johnson syndrome
3	Molnupiravir	Phase I/II/III US-FDA issued a EUA	G1	100%		Not recommended for pregnant and old-age patients
			G2	100%		
			G3	100%		
			G4	Can be used with caution		May impair bone and cartilage formation so it should not be given to patients <18 years of age
			G5	No dose recommendation is possible		
			G5D	No dose recommendation is possible		
			Risk for nephrotoxicity			A single patient of 67-year of age was found with nephrotoxicity. Not reported in other studies
			Risk for accumulation and systemic toxicity			Not reported
4	Oseltamivir	Phase III/IV	G1	100%		Adverse events include skin peeling, loosening of the skin, blistering, skin ulcers, and nose bleeding
			G2	100%		
			G3	100%		
			G4	100%		
			G5	A low dose is recommended		
			G5D	A low dose is recommended		
			Risk for nephrotoxicity			Not reported
			Risk for accumulation and systemic toxicity			Not reported
5	Azvudine	Phase III	G1	100%		Dizziness and nausea in the approx. 10% of patients
			G2	100%		
			G3	100%		
			G4	Lower dose is recommended		
			G5	Can be given with caution		
			G5D	Can be given with caution		
			Risk for nephrotoxicity			Not reported
			Risk for accumulation and systemic toxicity			Not reported
6	Famciclovir		G1	100%		Headache, nausea, stomach pain, tiredness, sleepiness and diarrhea
			G2	100%		
			G3	Multiple lower dose is recommended		
			G4	Lower dose is recommended		
			G5	Single low dose is recommended		
			G5D	Single low dose with caution is recommended		
			Risk for nephrotoxicity			Not reported
			Risk for accumulation and systemic toxicity			Not reported
7	Darunavir	Phase III/IV	G1	100%		
			G2	100%		
			G3	100%		
			G4	100%		
			G5	Can be given with caution		
			G5D	Can be given with caution		
			Risk for nephrotoxicity			When given along with cobicistat it should be avoided in patients with GFR <70 ml/min. Produced AKI in a single COVID-19 infected patient. Relatively safe
			Risk for accumulation and systemic toxicity			In case of mild liver dysfunctions, caution is advised without any dose modifications. In case of severe liver dysfunction, should be avoided
8	Dolutegravir/Rilpivirine	Phase I/IV (against HIV)	G1	100%		It may affect the level of drugs eliminated *via* OCT2 and MATE1 such as dofetilide and dalfampridine
			G2	100%		
			G3	100%		
			G4	100%		
			G5	Can be given with caution		
			G5D	Can be given with caution		It may cause dizziness and nasal congestion
			Risk for nephrotoxicity			Not reported
			Risk for accumulation and systemic toxicity			Not reported
9	Sofosbuvir	Phase II/III//IV	G1	100%		Sofosbuvir should not be given along with rifabutin and carbazepine
			G2	50%		
			G3	No dose recommendation possible		Fatigue, headache, and rash may be observed
			G4	Not recommended		
			G5	Not recommended		
			G5D	Not recommended		
			Risk for nephrotoxicity			Can induce AKI in patients having moderate to severe kidney impairment
			Risk for accumulation and systemic toxicity			Female patients and female spouses of male patients receiving any sofosbuvir-containing therapy should avoid pregnancy for up to 6 months after treatment ends
10	Nirmatrelvir/ritonavir combination	Phase I	G1	100%		Should be avoided along with calcium channel blockers, statins, and anti-coagulants
			G2	100% Can be given with caution		
			G3	50%		Dysgeusia, Diarrhea, HTN Myalgia
			G4	Not recommended		
			G5	Not recommended		
			G5D	Not recommended		
			Risk for nephrotoxicity			Not reported with eGFR of G3 phase. Shall be avoided in patients having G4, G5 eGFR.
			Risk for accumulation and systemic toxicity			May accumulate in the patients having mild-to-moderate kidney disease Not recommended with severe hepatic impairment
11	Favipiravir	Phase II/III	G1	100% can be given with caution		Tends to raise uric acid excretion
			G2	100% can be given with caution		GIT related problems
			G3	No dose recommendation is possible		
			G4			
			G5			
			G5D			
			Risk for nephrotoxicity			May cause acute kidney dysfunction
			Risk for accumulation and systemic toxicity			May cause liver enzyme abnormality Accumulates in patients with renal impairment
12	Ribavirin	Phase II	G1	100%		The dose should be selected with consideration of anaemia
			G2	100%		
			G3	50% can be given with caution		Decrease in hemoglobin
			G4	A low dose can be given with caution		Hemolysis
			G5	Not recommended		
			G5D	Not recommended		
			Risk for nephrotoxicity			Dose adjustment and continuous monitoring is required in kidney-impaired patients
			Risk for accumulation and systemic toxicity			Shows teratogenic effects if administered during pregnancy or breast feeding
13	Umifenovir	Phase IV	G1		Can be given with caution	In CKD and immunocompromised patients, the use of umifenovir is not suitable
					Not recommended	
			G2			

			G3			

			G4			

			G5			

			G5D			
						Nephrotoxicity frequent
			Risk for nephrotoxicity			
						No major side effects
			Risk for accumulation and systemic toxicity			
14	Lopinavir/ritonavir combination	Phase II/III/IV	G1	Can be given for a short time with caution		Diarrhoea, headache, stomach upset, and drowsiness are common
			G2	Not recommended		
			G3			
			G4			
			G5			
			G5D			
			Risk for nephrotoxicity			Associated with tubular damage and shows kidney injury after prolonged administration
			Risk for accumulation and systemic toxicity			Hepatotoxic, shall be avoided in patients having liver or kidney problems

Note* G1: GFR ≥90 ml/min/1.73 m^2^; G2: GFR 60–89 ml/min/1.73 m^2^; G3: GFR 30–59 ml/min/1.73 m^2^; G4: GFR 15–29 ml/min/1.73 m^2^; G5: GFR <15 ml/min/1.73 m^2^; G5D: GFR <15 ml/min/1.73m^2^ + dialysis.

Abbreviations* COVID-19: Coronavirus Disease-2019; CKD: chronic kidney disease; US-FDA: united states food and drug administration; EUA: emergency use authorization; i.v.: Intravenous; GFR: glomerular filtration rate; AKI: acute kidney injury; GIT: gastrointestinal; ALT: alanine aminotransferase; AST: aspartate aminotransferase; HSR: aypersensitivity reactions.

## 2 Antivirals for the treatment of COVID-19

### 2.1 RNA-dependent RNA polymerase (RdRp) inhibitor

#### 2.1.1 Remdesivir

**FIGURE 1:**
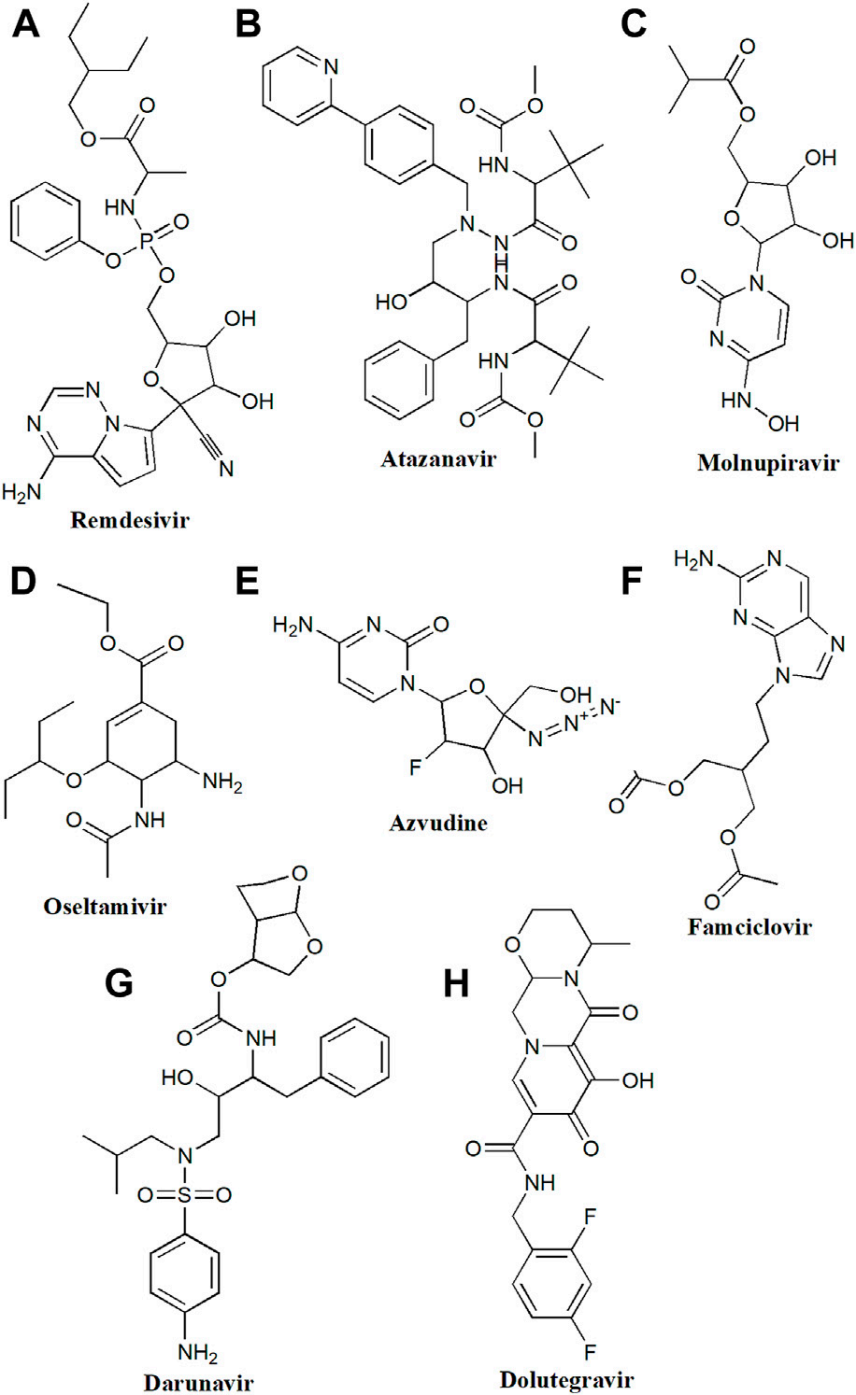
Chemical structure of **(A)** Remdesivir, **(B)** Atazanavir, **(C)** Molnupiravir, **(D)** Oseltamivir, **(E)** Azvudine, **(F)** Famciclovir, **(G)** Darunavir, **(H)** Dolutegravir.

Remdesivir is a broad-spectrum antiviral effective against RNA viruses such as Ebola, Middle East Respiratory Syndrome Coronavirus, and severe acute respiratory syndrome-CoV. In May 2020, the US Food and Drug Administration (US-FDA) issued an emergency use authorization (EUA) to permit remdesivir which acts *via* inhibition of RNA-dependent RNA polymerase (RdRp) (Grein et al., 2020). RdRp is a crucial polymerase required for replicating the single-stranded RNA genome of SARS-CoV-2. Remdesivir is a prodrug of adenosine triphosphate analog metabolized to remdesivir triphosphate, which competes with adenosine triphosphate for incorporation by RdRp and interferes with viral RNA replication (Figure 1A) (Beigel et al., 2020). Currently, remdesivir is recommended for hospitalized COVID-19 patients (Beigel et al., 2020). As per the revised Infectious Diseases Society of America (IDSA) and WHO guidelines, in patients with mild-to-moderate COVID-19 infection, remdesivir can be given at a dose of 200 mg i.v. on the first day followed by 100 mg for the next two or 3 days and for patients with severe COVID-19 two up to 10 days (https://apps.who.int/therapeutics). In adults with eGFR ≥60 ml/min/1.73 m2, 4 mg/kg, orally daily is recommended. For pediatric patients, 5 mg/kg on day one followed by 2.5 mg/kg is recommended (https://www.idsociety.org/COVID19guidelines). It should be administered as early as possible or within 7 days of COVID-19 onset and precautions must be taken in patients with liver or kidney disease (https://apps.who.int/therapeutics).

##### 2.1.1.1 Pharmacokinetic profile

Intravenous (i.v.) administration of remdesivir shows a better bioavailability compared to the intramuscular (i.m.) route. However, oral administration involves a hydrolysis-mediated first pass clearance in the gastrointestinal tract (Deb et al., 2021). The parent remdesivir has the highest affinity for human plasma proteins with 88–93.6% binding than its metabolized forms (1–2%). Upon hydrolysis, remdesivir converts to the active triphosphate form (GS-443902). Remdesivir has a volume of distribution of about 45.1–73.4 L with a single dose of 10 mg–225 mg, whilst frequent dosing for a couple of weeks showed 85.5 L (Humeniuk et al., 2020). Moreover, the instability of remdesivir is responsible for its low volume of distribution. To achieve an optimum drug concentration in infected tissue, frequent dosing of the drug is necessary. However, doses of remdesivir of more than 200 mg can cause hepatotoxicity and nephrotoxicity, indicating a safety threshold of remdesivir (Sun, 2020). Due to the high extraction ratio and shorter elimination half-life, it is estimated that hepatic clearance of remdesivir is mainly affected by hepatic blood flow and merely by its metabolism (Deb et al., 2021). Parent remdesivir and GS-704277 (alanine metabolite) are majorly excreted *via* biotransformation, however, GS-441524 is eliminated by renal excretion. Approximately, out of the total dose of remdesivir, 10% of the parent form and 49% of the metabolite form- GS-441524 are excreted *via* the urine (Humeniuk et al., 2020; Deb et al., 2021; Humeniuk et al., 2021)**.**

##### 2.1.1.2 Rationale for use in COVID-19

Remdesivir is recommended for severe and hospitalized COVID-19 patients based on the results of a series of trials such as NCT04292899, NCT04321616, NCT04330690, and NCT04365725 (Grein et al., 2020; WHO Solidarity Trial Consortium, 2022). Remdesivir shortens the time of recovery from COVID-19 and lowers respiratory tract-related complications due to COVID-19. However, little is known about the use of remdesivir in COVID-19 patients with CKD (Thakare et al., 2021). CKD patients with COVID-19 have higher viral loads of SARS-CoV-2 compared to patients without CKD, hence the normal dose range does not produce optimal effects (Ortiz, 2021). The remdesivir carrier sulfobutylether-β-cyclodextrin (vehicle for intravenous injection) can accumulate in the kidney tubules and cause nephrotoxicity (Hafner et al., 2010). The normal remdesivir dose contains 3–6% of sulfobutylether-β-cyclodextrin, which is more than its recommended dose of 250 mg/kg per day (Luke et al., 2012). Interestingly, choosing the nasal route for the administration of remdesivir would avoid direct nephrotoxicity in COVID-19 patients with CKD (Thakare et al., 2021). In general, antivirals can cause mitochondrial injury in kidney epithelial cells however, remdesivir seems to have a very low potential to cause mitochondrial toxicity (Sun, 2020). Interestingly, the outcomes of a recent clinical trial (NCT04292730) on remdesivir revealed that the percentage of risk of kidney malfunction was less than 1% and it did not increase the risk of renal adverse events (Grein et al., 2020). Thus, remdesivir can be used in COVID-19 patients with CKD (with GFR >30) wherein the preferred route would be nasal (Table 1). However, more clarification is needed for remdesivir in end-stage kidney disease (ESKD).

##### 2.1.1.3 Limitations and adverse effects

Remdesivir at a dose of 5, 10, or 20 mg/kg induced kidney injury in rhesus monkeys (Warren et al., 2016). Interestingly, no kidney-related adverse events have been found in clinical trials of COVID-19 and Ebola (Mulangu et al., 2019).Nephrotoxicity may rather relate to the vehicle sulfobutylether-β-cyclodextrin and not necessarily to remdesivir itself. Thus, remdesivir injections in patients with severe kidney dysfunction are problematic. Pharmacokinetic studies of remdesivir were not reported in kidney disease patients. The EUA fact sheet suggests that remdesivir is not recommended for pediatric patients and neonates with eGFR less than 30 ml/min and creatinine clearance ≥1 mg/dl unless the possibility of benefit is more than the potential risk (US Food and Drug Administration, 2021). Other adverse events like nausea, elevation in alanine aminotransferase (ALT) and aspartate aminotransferase (AST) levels, and prolongation in prothrombin time is reported. Hence, considering the aforementioned studies and all the pharmacokinetic aspects of remdesivir, it is safe, effective, and can be used at recommended dose against COVID-19 patients with kidney impairment (GFR having >30) (Table 1).

#### 2.1.2 Molnupiravir

Molnupiravir is a prodrug of ribonucleoside beta D-N4-hydroxycytidine (NHC) with significant antiviral activity for RNA viruses including influenza and coronavirus (Figure 1C) (Singh et al., 2021). Molnupiravir received a EUA from the US FDA in December 2021 to treat adults with moderate COVID-19 infection and those who were at high risk for developing severe illness within 5 days of symptom onset (Fischer et al., 2021). The existing recommended dose for moderate to severe COVID-19 patients is 800 mg p.o. per 12 h for 5 days (https://apps.who.int/therapeutics). Various clinical trials evaluated its safety, tolerability, and pharmacokinetics in healthy individuals as well as in COVID-19 patients (NCT04392219, NCT04746183, NCT04405570, NCT04405739, and NCT04575597).

##### 2.1.2.1 Pharmacokinetic profile

As a prodrug, after 0.5 and 1 h of administration, molnupiravir is observable at very low quantities (Khoo et al., 2021). NHC rapidly changes intracellularly to its phosphorylated form, NHC-TP upon oral administration of 1^st^ dose in humans, resulting in quick absorption (Tmax-1.0–1.75 h) but shorter plasma half-life (t1/2–1 h). A biphasic clearance occurs following repeated administration of NHC at clinical doses along with increased mean t1/2–7.08 h (Painter et al., 2021). Additionally, no rise in serum concentration of molnupiravir was seen following single or multiple dose treatments (Yoon et al., 2018; Toots et al., 2019). No detectable protein binding of molnupiravir is observed, however, it possesses a limited volume of distribution around 142 L (Food and Drug, 2022). Molnupiravir does not show significant metabolism once converted to NHC-TP. Little NHC is excreted *via* the urine in unchanged form. Following single or multiple-doses, approximately 0.85% and 3.61% of the doses are excreted into the urine (Food and Drug, 2022). Hence, renal clearance does not play a significant role in molnupiravir removal from the body. This distinguishes molnupiravir from other nucleoside analogues currently available.

##### 2.1.2.2 Rationale for use in COVID-19

As NHC, the active form of Molnupiravir is not significantly eliminated by kidneys, patients with any degree of kidney dysfunction do not require dosage adjustments (Table 1) (Food and Drug, 2022). Mild or moderate kidney dysfunction had no appreciable effect on molnupiravir pharmacokinetics (Painter et al., 2021). Even though the pharmacokinetics of NHC has not yet been investigated in patients with an eGFR <30 ml/min/1.73m2 or on dialysis these should not have a considerable effect on NHC clearance (Food and Drug, 2022). Among the unvaccinated adults with COVID-19, molnupiravir reduced the risk of hospitalization and death rates (NCT04575597).

##### 2.1.2.3 Limitations and adverse effects

A 67-year-old patient developed acute tubular necrosis upon molnupiravir treatment. However, kidney function improved with intravenous hydration (Yücel, 2022). Apart from this single case, molnupiravir seems found safe and hence no dose adjustment is required in patients with kidney and/or hepatic dysfunction (Singh et al., 2022a). Molnupiravir should not be prescribed during pregnancy considering the embryo-fetal toxicity reported in pre-clinical settings (Singh et al., 2021; Singh et al., 2022b). Also, for as long as a person with child-bearing potential is exposed to NHC systemically, it is suggested to use contraceptives (During and after 4 days of treatment) (Singh et al., 2022b). Similarly, preclinical studies have also shown that molnupiravir may impair bone and cartilage formation so it should not be given to patients <18 years of age. Other common side effects include diarrhea, nausea, and dizziness (Singh et al., 2022b; Food and Drug, 2022) (Table 1).

#### 2.1.3 Famciclovir

Famciclovir is a prodrug and a nucleoside analog effective against herpes simplex virus infections caused by varicella zoster (chickenpox) and herpes zoster (Figure 1F) (Yoon and Rhew, 2013). Treatment with famciclovir can decrease the spread of viral infection from one organ to another organ in the body.

##### 2.1.3.1 Pharmacokinetic profile

The pharmacokinetics of famciclovir have been studied more than 3 decades ago (Pue and Benet, 1993). Famciclovir is an oral prodrug quickly metabolized to active metabolite penciclovir and reaches therapeutic plasma concentrations within 1 h of oral administration (Pue and Benet, 1993). Interestingly, the volume of distribution is more after i.v. administration i.e., 1 L/kg. However, the plasma concentration is affected by the presence of food. However, the bioavailability is not affected by food and is measured by a urinary recovery which is approx. 60%. The major route of elimination of penciclovir is the kidney. Penciclovir is extensively cleared within 2–2.5 h and renal clearance lies between 25 and 30L/h in normal patients. The clearance rate of this drug is solely dependent on kidney function. Therefore, patients with kidney impairment (CKD and ESKD) require more time to clear penciclovir (Pue and Benet, 1993; Gill and Wood, 1996). The reported dose for famciclovir lies between 125 and 1500 mg per day depending on the status of kidney function.

##### 2.1.3.2 Rationale for use in COVID-19

COVID-19-associated pneumonia involves cell-mediated inflammation (CMI) (Shiraki et al., 2022). Interestingly, herpes zoster virus infection also causes adaptive CMI mediated by CD4 and CD8 lymphocytes and cytokines. Shiraki and colleagues observed that famciclovir is very effective against inflammatory lesions caused by adaptive CMI in moderate herpes zoster (Shiraki et al., 2021; Shiraki et al., 2022). Famciclovir accelerates viral clearance and prevents swelling and vesiculation. Nevertheless, the effect of famciclovir on COVID-19 has been explored less. A 58-year-old woman with meningitis and COVID-19 was treated with famciclovir for 6 days and showed a stable condition (Packwood et al., 2020). Famciclovir is proven safe in patients with kidney impairment. One case report shows that famciclovir gradually improves kidney function more than aciclovir in patients with initial kidney dysfunction (Yoon and Rhew, 2013). Also, the dose of famciclovir can be adjusted based on the creatinine clearance rate of patients with recurrent herpes labialis (APO-FAMCICLOVIR 125, 250, and 500 mg film-coated tablets, Apotex New Zealand, New Zealand Data Sheet) for example, no dose adjustment (1500 mg single dose) is required for those patients with creatinine clearance >60 ml/min/1.73 m^2^, similarly, the clearance 20–39 ml/min/1.73 m^2^ can be treated with 500 mg single dose. Patients on hemodialysis can be treated with a 250 mg single dose (Table 1). Overall studies demonstrate that famciclovir might prove beneficial to treat COVID-19-infected CKD and ESKD patients.

##### 2.1.3.3 Limitations and adverse effects

Patients on hemodialysis might require special attention while treated with famciclovir. 4h of hemodialysis can clear up to 75% penciclovir from the blood, therefore, famciclovir should be administered immediately following dialysis.

Importantly, the major excretion route for this drug is the kidney hence, famciclovir accumulates in patients with impaired kidney function. Thus, dose adjustments are necessary for CKD patients (Table 1). Common side effects include headache, nausea, stomach pain, tiredness, sleepiness, and diarrhoea (Yoon and Rhew, 2013).

#### 2.1.4 Ribavirin

**FIGURE 2:**
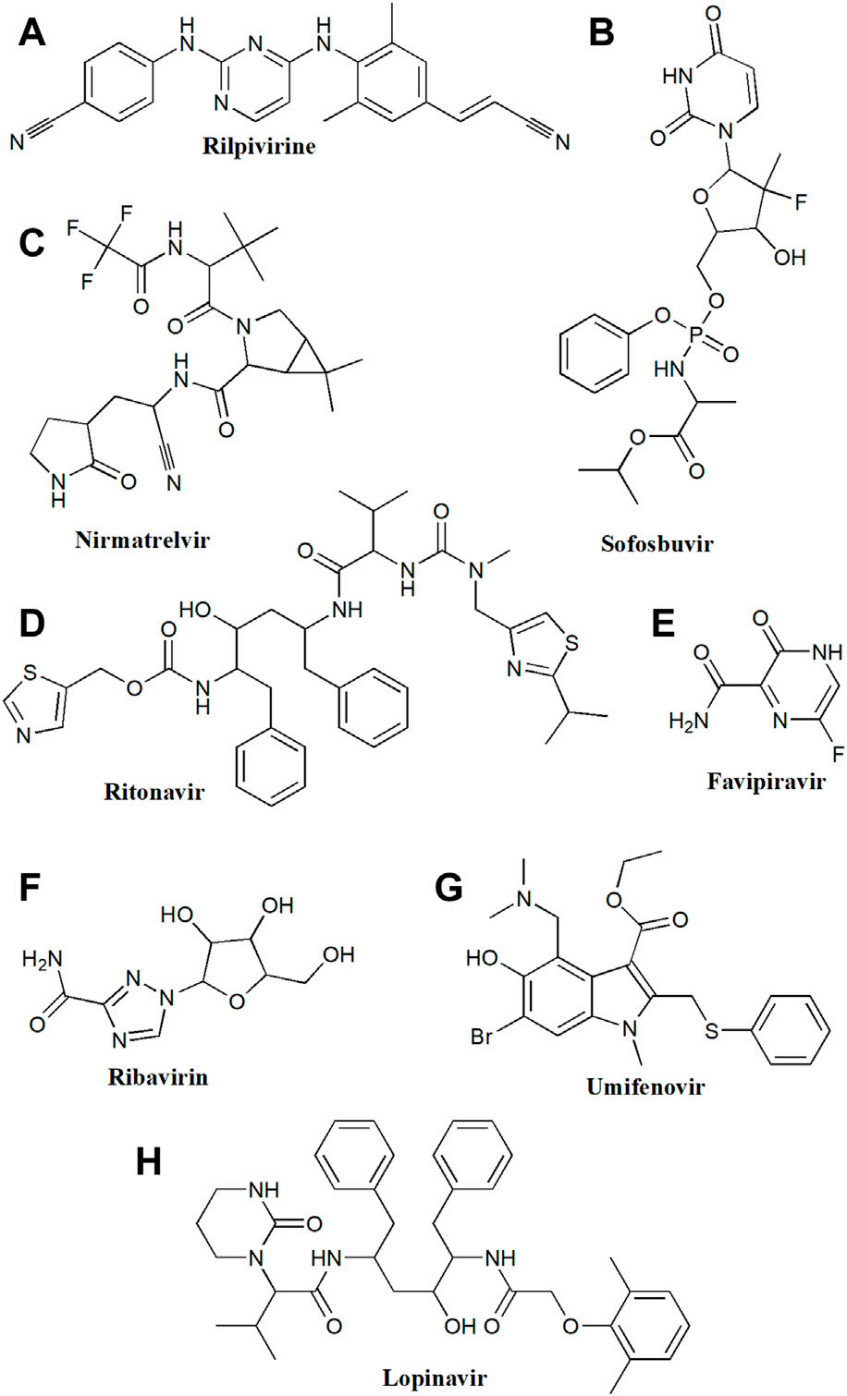
Chemical structure of **(A)** Rilpivirine, **(B)** Sofosbuvir, **(C)** Nirmatrelvir, **(D)** Ritonavir, **(E)** Favipiravir, **(F)** Ribavirin, **(G)** Umifenovir, **(H)** Lopinavir.

Ribavirin, a guanosine analogue is certified to treat hepatitis C along with pegylated interferon 2a or 2b or directly acting antiviral drugs (Figure 2F). It has shown antiviral action against SARS-CoV-2 *in-vitro* by interfering with RNA translation and viral multiplication (Tarighi et al., 2021). Ribavirin is administered orally and is medicated as per the weight, varying from 800 mg to 1200 mg daily in multiple small doses for hepatitis C treatment (Hung, 2020). Different doses of ribavirin are investigated in COVID-19 patients, including 400 mg twice daily when taken with lopinavir/ritonavir (LPN/r) or 500 mg two or three times per day by oral or i.v. route (Hung, 2020; Khalili et al., 2020). For mild-to-moderate COVID-19, the combination of ribavirin with LPN/r and IFN-β-1b contributed to shorter hospitalization as compared to LPN/r monotherapy (Hung et al., 2020). But there are not enough shreds of evidence to support its significant contribution to lowering the mortality rate as clinical trials are still going on (NCT04494399).

##### 2.1.4.1 Pharmacokinetic profile

According to the pharmacokinetic investigations in healthy individuals administered with 150 mg of 13C3-ribavirin i.v. accompanied by a 400 mg oral dose 1 h later, ribavirin showed a mean bioavailability of 52% ± 22% along with a mean half-life of around 37 ± 14 h (Preston et al., 1999). Ribavirin concentrations take around 4 or more weeks to stabilize because of their high volume of distribution and its excretion which relies on kidney function. Ribavirin is phosphorylated intracellularly into mono, di-, and triphosphates resulting in its activation (Morello et al., 2008). In volunteers with healthy kidney function taking ribavirin doses as per their body weights for hepatitis C infection- 1g (<75 kg body weight) or 1.2 g per day (≥75 kg body weight) categorized into 2 oral doses, around 8–12 μmol/L of plasma ribavirin concentration are attained at steady-state (Waldenström et al., 2016). Although the ideal ribavirin target peak concentration is still unknown, it should be underlined that at plasma levels higher than 15 μmol/L, toxic effects rise noticeably (Lagging et al., 2017). Despite this, significantly larger doses of ribavirin are indicated for several viral infections such as Lassa hemorrhagic fever (Bausch et al., 2010).

##### 2.1.4.2 Rationale for use in COVID-19

Ribavirin is primarily excreted by the kidneys (62%), and individuals with altered kidney functions have significantly changed the pharmacokinetics of ribavirin. Though ribavirin is used to treat a large number of individuals with kidney impairment or those undergoing hemodialysis with deteriorated kidney function can worsen the associated toxicities (anaemia). The dose of ribavirin should be selected with consideration of anaemia (reduced dose with low baseline haemoglobin) **(**Roche. COPEGUS^®^ (ribavirin) Tablets, US Prescribing Information). With decreased creatinine clearance, ribavirin plasma concentration rises. The well-tolerated dosage modifications for kidney impairment result in approximate equivalent ribavirin plasma concentration as that in subjects without kidney dysfunction (Brown and Wobst, 2021). As hemodialysis does not eliminate ribavirin, so ribavirin dose should be minimized for kidney-impaired patients or those taking RRT (Rebetol 200 mg hard capsules Summary of Product Characteristics. 2019) (Table 1).

##### 2.1.4.3 Limitations and adverse effects

Ribavirin is contraindicated in patients with severe kidney failure (Lexi-comp, 2018). Also, ribavirin administration should be stopped immediately if serum creatinine levels in children increase by more than 2 mg/ml (Wang and Zhu, 2020). Ribavirin is not recommended for use during pregnancy as well as during breastfeeding due to the possible teratogenic effects observed in pre-clinical settings (Sinclair et al., 2017). However, preliminary investigations of the ‘Ribavirin Pregnancy Registry’ have not yet been able to prove any conclusive proof for ribavirin’s human teratogenic effects (Sinclair et al., 2017). Therefore, it is advised that women treated with ribavirin should avoid pregnancy even after 4 months after treatment, and female partners of treated men should avoid pregnancy for 7 months after ribavirin exposure (Sinclair et al., 2017). Ribavirin also known to decrease hemoglobin level, to cause hemolysis and anemia (Giampaoli et al., 2022). Hence, monitoring of hematological parameters is advisable in patients’ taking ribavirin.

#### 2.1.5 Favipiravir

Favipiravir, a broad-spectrum antiviral medication is a purine nucleic acid analogue that has been approved in Japan to treat influenza (Figure 2E). The active metabolite, favipiravir ibofuranosyl-5′-triphosphate (T-705-RTP) of prodrug favipiravir is generated by its ribosylation and phosphorylation (Furuta et al., 2013). The incorporation of T-705-RTP into the viral RNA hinders viral replication by competing with purine nucleotide thereby, resulting in the suppressed activity of RdRp (Furuta et al., 2013). For influenza treatment, favipiravir is administered 1600 mg twice daily (Day1) and 600 mg twice daily (Day2–5) (Du and Chen, 2020). The outcome of several completed clinical trials conducted for evaluating the efficacy and safety of favipiravir against COVID-19 showed promising outcomes (NCT04351295, NCT04402203, NCT04542694, and NCT04448119). Favipiravir is explored at several doses for treating COVID-19, including a maintenance dose of 200–600 mg twice daily for 10–14 days and various loading doses of 1600, 1800, and 2400 mg (Chen et al., 2020).

##### 2.1.5.1 Pharmacokinetic profile

As per the studies conducted on healthy Japanese subjects, favipiravir reached its peak plasma concentration 2 h after its oral administration and subsequently showed a rapid decline with a short half-life of 2–5.5 h (Madelain et al., 2016). In humans, Favipiravir is bound to plasma proteins at a 54% rate, and its observed binding with human serum albumin as well as with α1-acid glycoprotein was 65% and 6.5%, respectively (Mentré et al., 2015). Favipiravir is metabolized by aldehyde oxidase and xanthine oxidase in the liver to produce the inactive metabolite T-705M1, which is then eliminated by the kidneys (Madelain et al., 2016).

##### 2.1.5.2 Rationale for use in COVID-19

The excretion of favipiravir is through the kidneys (90%), out of which M1 accounts for 82–92.4% of the total. In the phase-III clinical trial of investigation, 30 patients with an eGFR of 50–80 ml/min were included, only 1 patient with an eGFR of 30–50 ml/min, and no patients with eGFR less than 30 ml/min were included. There is no documented pharmacokinetic data for the population with an eGFR of 50–80 ml/min (Du and Chen, 2020). In patients with mild to moderate renal impairment, the M1 levels were elevated 2.5 times; however, it is based on one patient’s favipiravir clearance. Given that favipiravir accumulates in patients with renal impairment, any potential toxicity is most likely attributed to M1 (Du and Chen, 2020). However, there is insufficient information to draw any conclusion related to its safety in individuals with renal impairment or those relying on dialysis. Furthermore, as stated by Mishima et al., favipiravir tends to raise urine uric acid levels, which needs to be explored in renal impairment patients. A total of 13/30 patients (43.4%) with mild renal dysfunction and 110/363 (30%) patients with normal kidney function experienced adverse events in a phase-III study, which had a brief treatment duration equal to that used in COVID-19 (Mishima et al., 2020). Altogether, there is not enough evidence to recommend the use of favipiravir for individuals with kidney dysfunction or those undergoing dialysis (Table 1). There is no evidence of clinical benefits from favipiravir in COVID-19 patients without kidney dysfunction (Bosaeed et al., 2022; Shah et al., 2022).

##### 2.1.5.3 Limitations and adverse effects

Favipiravir appears to be safe in terms of overall and serious adverse events as per the existing clinical reports. However, as shown by a meta-analysis of larger trials, elevations in blood uric appear to be a safety issue, with some indications of an escalating dose-dependent pattern. It is also reported for modifying the levels of liver enzymes and to cause gastrointestinal discomfort. The possibility of teratogenicity and QTc prolongation has not been investigated yet (Pilkington et al., 2020). The safety and tolerance of favipiravir in short-term usage are supported by data but further information is required to evaluate its long-term effects. As per the findings of a case report, non-oliguric AKI was observed after 48 h of favipiravir administration in two COVID-19 patients having normal baseline creatinine clearance. Moreover, kidney damage was improved within 24–48 h of therapy discontinuation (Nasa et al., 2021). Therefore, the use of favipiravir for COVID-19 patients having kidney impairment is indicated with caution due to the limitations of the evidence and persisting safety concerns (Pilkington et al., 2020).

### 2.2 Protease inhibitors

#### 2.2.1 Atazanavir

Atazanavir (ATV) belongs to the class of protease inhibitors and is an azapeptide that preferentially suppresses the activity of HIV-I protease, a key enzyme involved in the maturation of HIV-1 virions (Figure 1B) (De Clercq and Li, 2016). It is approved by FDA to treat HIV infection either alone (400 mg) or by combining with pharmacokinetic enhancer ritonavir (300/100 mg). The drug is currently tested in clinical trials at different phases for COVID-19 (NCT04468087-II/III, NCT04452565-II/III, and NCT04459286-II). The dose suggested for treating COVID-19 is identical to that for HIV infection (REYATAZ (atazanavir) capsules, US Prescribing Information. BMS). ATV (300 mg/day *p.o.*) when administered with other antivirals improved oxygen saturation, clinical and paraclinical characteristics in COVID-19 patients (Kalantari et al., 2021).

##### 2.2.1.1 Pharmacokinetic profile

ATV is quickly absorbed and reaches its highest concentration (Cmax) after 2–2.5 h of administration. It is recommended to administer ATV with food for increased absorption (Busti et al., 2004). The fraction-bound distribution of ATV is 86%. The cytochrome P450 family 3 subfamily A (CYP3A) polypeptide isoenzymes 4 and 5 (CYP3A4/5) are predominantly responsible for the metabolism of ATV (Tiec et al., 2005). Individuals possessing a minimum of one functioning copy of CYP3A5 experience mono-oxidation and oral clearance of ATV (unboosted) more quickly than those lacking any functional versions attributed to homozygosity of CYP3A5*3, CYP3A5*6, or CYP3A5*7 (Wempe and Anderson, 2011; Castillo-Mancilla et al., 2016). As bile accounts for excreting the majority of ATV’s prescribed dose (79% unchanged) and only around 13% is eliminated as metabolites in urine (Tiec et al., 2005). So, ATV therapy can be given to patients with impaired kidneys with special monitoring (Table 1).

##### 2.2.1.2 Rationale for use in COVID-19

ATV can be used to treat COVID-19 in patients with kidney impairment at the regular dosage of 400 mg (unboosted) or 300/100 mg (boosted) because of its negligible renal clearance (EACS Guidelines version 10.0. November 2019). It was stated earlier that its sensitivity/exposure was reduced by around 30–50% in subjects who received hemodialysis (Reyataz 300 mg Hard Capsules, Summary of Product Characteristics. Last updated on EMC: 25 February 2019, BMS). Nonetheless, one case study found that individuals receiving dialysis and those who were not on dialysis had comparable ATV elimination (Izzedine et al., 2005). To ensure appropriate exposure in RRT-receiving subjects, boosted ATV may be chosen due to limited information presently available and the possibility of elevated ATV clearance during and after hemodialysis (Izzedine et al., 2005).

##### 2.2.1.3 Limitations and adverse effects

Chronic exposure to ATV can lead to granulomatous interstitial nephritis and glomerulosclerosis (Varghese et al., 2020). However, the drug still can be prescribed for COVID-19 patients with CKD as the renal elimination of ATV is very less and also treatment duration during COVID-19 is shorter. Thus, it should not be prescribed as chronic therapy and in patients with severe CKD or ESRD. Further, as ATV is metabolized by CYP3A4 which also inhibits CYP1A2, CYP3A4, and CYP2C9, patients taking medications with a restricted therapeutic index should avoid ATV administration (Choi et al., 2020). Warfarin, simvastatin, irinotecan, lovastatin, and phosphodiesterase inhibitors are a few examples of drugs that may have substantial drug interactions with ATV (Choi et al., 2020). Sometimes, ATV can cause allergic reactions that manifest as a moderate rash, toxic skin eruptions, erythema multiforme, or Stevens-Johnson syndrome (Choi et al., 2020). Patients should stop ATV administration if they experience severe hypersensitivity reactions (Table 1).

#### 2.2.2 Darunavir

Darunavir (DRV), an HIV protease inhibitor limits the maturation of infectious virions by interrupting the HIV-encoded Gag-Pol proteins in the cells (Figure 1G) (Roberto et al., 2020). It has demonstrated a strong binding affinity for the SARS-CoV-2 protease in *in silico* studies (Milburn et al., 2017). DRV is recommended with booster cobicistat to bypass the inhibition of DRV by cytochrome P450 (Gutierrez‐Valencia et al., 2018). Various clinical investigations (NCT04425382, NCT04252274) suggest that darunavir/cobicistat (DRV/c) could be used as an effective option for COVID-19 treatment (Milburn et al., 2017). The recommended dose of DRV/c is 800/150 mg once daily for 5–7 days (Roberto et al., 2020). The mode of action of DRV/c is similar to that of LPN/r; hence can be used as a substitute for COVID-19 treatment when LPN/r is unavailable (Roberto et al., 2020).

##### 2.2.2.1 Pharmacokinetic profile

When taken orally, DRV is quickly absorbed. A single 600 mg dose of DRV monotherapy has an optimum bioavailability of about 37% (Koh et al., 2007). As per preclinical studies, DRV shows 95% binding to α1-acid glycoproteins and up to some extent with albumin (Kakuda et al., 2014). The metabolism of DRV/c takes place in the liver by CYP450 (Roberto et al., 2020). In situations of mild liver dysfunctions, caution is advised without any dose modifications. However, in case of severe liver dysfunction, DRV/c administration should be avoided. DRV is minimally cleared by the kidneys (Roberto et al., 2020). In faeces and urine, unchanged DRV is nearly 41.1 and 7.7% of the given dosage, respectively (Koh et al., 2007).

##### 2.2.2.2 Rationale for use in COVID-19

DRV similar to LPN/r has a low elimination *via* the kidneys and has a high protein binding rate, which makes it difficult to remove by dialysis (Roberto et al., 2020). No dose adjustments are required for patients with kidney impairment (Table 1). Cobicistat is rather avoided in individuals with GFR <70 ml/min because it can impede kidney clearance (Table 1) (Roberto et al., 2020). Lastly, there is no information or clinical research documented regarding the dialysis clearance of DRV/c.

##### 2.2.2.3 Limitations and adverse effects

Crystal-induced nephropathy and proximal tubulopathy were observed in a 61-year old HIV patient treated with combination therapy including DRV and tenofovir (Soto et al., 2019). Similarly, a case report showed the incidence of acute kidney injury by DRV in a COVID-19 patient (Abdalla et al., 2020). This could be because of the poor solubility of DRV in urine. This potential complication should be considered during prescribing DRV therapy to patients having any degree of kidney impairment. Vomiting, eczema, diarrhea, peripheral neuropathy, and lipodystrophy are some other adverse events of DRV that are frequently reported. Altered heart rate and impaired liver function account for some rare adverse events after DRV therapy (Marin et al., 2021).

#### 2.2.3 Nirmatrelvir/ritonavir combination

Nirmatrelvir and ritonavir are another protease inhibitor combination effective against mild to severe SARS-CoV-2 infection (NCT05366192, NCT05341609, NCT05386472) (Figures 2C, D). A clinical trial is ongoing in COVID-19-infected adults with severe renal impairment (Phase I: NCT05487040). Fascinatingly, COVID-19 associated mortality rate was lowered by 89% after the treatment with nirmatrelvir (Hammond et al., 2022a). It acts by inhibiting the 3-chymotrypsin-like cysteine protease enzyme responsible for viral replication. The dose range for COVID-19 patients having eGFR ≥60 ml/min is 300 mg of nirmatrelvir and 100 mg of ritonavir twice a day for 5 days. In the patients having an eGFR ≥30–60 ml/min, nirmatrelvir is recommended at the dose of 150 mg with ritonavir at the dose of 100 mg twice daily for 5 days (https://www.covid19treatmentguidelines.nih.gov). However, it is not suggested for those with eGFR of <30 ml/min per 1.73 m^2^ (Table 1) (Hammond et al., 2022a).

Ritonavir represents a class of protease inhibitors used either alone or in combination to treat HIV infection. Ritonavir acts as a pharmacokinetic booster that inhibits the cytochrome 3A4 (CYP3A4) enzyme and is known for enhancing the pharmacokinetic properties of other antiviral drugs such as lopinavir, nirmatrelvir, and atazanavir.

##### 2.2.3.1 Pharmacokinetic profile

Ritonavir (100 mg) acts as a pharmacokinetic enhancer for other drugs such as nirmatrelvir and lopinavir (Wyatt et al., 2020). Preclinical studies have shown that the concentration threshold required for efficacy was 292 ng/ml indicating the dose should maintain this level, and therefore 300 mg dose was chosen for further clinical studies (Hiremath et al., 2022). Oral administration of nirmatrelvir shows rapid absorption with *t*_max_ for more than 3 h. A single oral dose of nirmatrelvir (300 mg) shows C_max_ 2.21 μg/ml with AUC inf 23.01 µg*h/mL. After absorption, nirmatrelvir distributed well in tissue (104.7 L). The protein binding is 69% and minimally metabolized by CYP3A4 (due to ritonavir). Moreover, ritonavir is metabolized in the liver, and hence mean concentration of nirmatrelvir is high even in kidney disease patients (eGFR <30 ml/min per 1.73 m^2^). Nirmatrelvir is mainly eliminated by renal excretion (approx. 35%) where the remaining is a protein-bound drug.

##### 2.2.3.2 Rationale for use in COVID-19

The drug had shown its potential against COVID-19 as it reduces lethality by 89%. It has been shown that treatment with nirmatrelvir–ritonavir reduces the risk of hospitalization or progression to severe COVID-19 infection in vaccinated patients with COVID-19 (Cao et al., 2022). The safety profile of nirmatrelvir is promising, as it has mild side effects and does not have dose-dependent toxicity. Moreover, clinically insignificant amounts can be cleared in patients requiring dialysis. Currently, the dose of nirmatrelvir is 300 mg with 100 mg ritonavir for those who have normal kidney function and 150 mg nirmatrelvir with 100 mg ritonavir can be given for those having 30–60 ml/min per 1.73 m^2^ (Hiremath et al., 2022). Interestingly, 300 mg nirmatrelvir with 100 mg ritonavir on the first day followed by 150 mg nirmatrelvir with 100 mg daily administration after hemodialysis may show good bioavailability (Brown et al., 2022). Also, a drug interaction is a challenge in CKD patients. Most of the treatment regimen for CKD includes calcium channel blockers, statins, and anti-coagulants. Therefore, little reduction in doses of drugs that follows CYP3A4 metabolism (as ritonavir inhibits CYP3A4) will not produce contradiction to treatment in CKD patients. However, close monitoring and dose adjustment for this combination is essential in CKD patients and patients on hemodialysis (Table 1).

##### 2.2.3.3 Limitations and adverse effects

No direct nephrotoxicity has been reported against nirmatrelvir. Preclinical studies suggest that nirmatrelvir is safe up to 1000 mg/kg per day which is 8 times more than the dose recommended for humans (Sathish et al., 2022).

With a molecular weight of 499 kD, the nirmatrelvir was majorly excreted through the kidney (35%) while approximately 70% remained as a protein-bound drug. Also, the nirmatrelvir accumulated with a decrease in kidney function. The dose adjustment is required for mild-to-moderate kidney disease patients having COVID-19 (Hammond et al., 2022b). Mild adverse events (Dysgeusia, Diarrhea, HTN, Myalgia) can be seen in critically ill patients (Hammond et al., 2022a). The combination is not recommended in severe liver dysfunction.

#### 2.2.4 Lopinavir/ritonavir combination

Lopinavir is a peptidomimetic protease inhibitor used in combination with ritonavir against HIV infection. Moreover, it is also effective against chronic hepatitis B or C. Initially, lopinavir was considered the drug of choice for COVID-19 based on preclinical trials. However, the later clinical studies revealed that the lopinavir/ritonavir combination shows insignificant effects against COVID-19 (Horby et al., 2020). This combination is also being studied with other antivirals against COVID-19 (NCT04307693, NCT04738045, NCT04499677). In a study of 3424 patients with COVID-19, there was no association between lopinavir–ritonavir treatment and the risk of 28-day mortality, length of hospital stay, or progression to invasive mechanical ventilation or mortality (Horby et al., 2020).

##### 2.2.4.1 Pharmacokinetic profile

The plasma concentration of lopinavir is rendered by the cytochrome 3A4 enzyme and hence ritonavir is used in combination which acts as a pharmacokinetic booster for lopinavir (Best et al., 2011). The pharmacokinetic open-label study in HIV-infected children showed that administration of two Kaletra^®^ tablets (Lopinavir/ritonavir 200/50 mg/kg respectively) in a day shows faster absorption (Best et al., 2011). The study found variation under the curve (AUC), Cmax, and C12, possibly due to age, sex, and weight difference. Nevertheless, high fatty food increases the AUC of lopinavir (26.9%). An increase in plasma clearance rate is directly proportional to the decrease in bioavailability and not due to faster elimination. Lopinavir is primarily cleared by the hepato-biliary route in animals and significantly *via* the urinary route in humans.

Unfortunately, lopinavir is directly associated with tubular damage and shows kidney injury after prolonged administration which eventually led to chronic kidney disease (Table 1). Therefore it should be taken for short period of time with continuous monitoring and/or may not be recommended for chronic therapy in COVID-19 patients having later stages of CKD (Jose et al., 2017; Binois et al., 2020).

##### 2.2.4.2 Rationale for use in COVID-19

This combination is prescribed frequently for general population of COVID-19. If a patient has CKD (GFR = 90 ml/min/1.73 m^2^), it can be prescribed for a short period of time with caution and continuous monitoring. However, in COVID-19 patients having GFR <89 ml/min/1.73 m^2^ it is not recommended to use. Moreover, it did not significantly reduce hospital stay or mortality rate among the COVID-19 patients.

##### 2.2.4.3 Limitations and adverse effects

Diarrhoea, headache, stomach upset, and drowsiness are common with lopinavir. Besides, lopinavir-induced acute kidney toxicity and hepatotoxicity are the prime concerns, thus, the use of this drug should be avoided in special populations having liver or kidney disease (Binois et al., 2020).

### 2.3 Neuraminidase inhibitors

#### 2.3.1 Oseltamivir

Oseltamivir is a neuraminidase inhibitor, recommended as a first-line treatment for influenza A and influenza B viruses (Figure 1D) (Zendehdel et al., 2022). In 2004, Zhang and colleagues found that the active site of S1 (spike 1) protein resembles the neuraminidase binding site of oseltamivir suggesting that oseltamivir might be beneficial in COVID-19 (Zhang and Yap, 2004). The recommended dose of oseltamivir is 75 mg once or twice a day for 5–14 days, alone or in combination with drugs like hydroxychloroquine or azithromycin in COVID-19 patients (NCT04516915, NCT04338698). The administration of oseltamivir in COVID-19 patients shorten hospital stays and also reduced the mortality rate (Zendehdel et al., 2022). 

##### 2.3.1.1 Pharmacokinetic profile

After oral administration, oseltamivir is rapidly converted into active metabolite i.e., oseltamivir carboxylate by hepatic esterase in GIT (Patel et al., 2015). This carboxylate form binds and inhibits active sites of neuraminidase enzyme in influenza. Oseltamivir carboxylate occurs in plasma within 30 min of administration and reaches C_max_ after 3–4 h. Interestingly, gastric acid pH, antacids, and food presence do not alter oseltamivir’s bioavailability (Kute et al., 2011). With a volume distribution of nearly 26 L, oseltamivir carboxylate can be available at the infected site with the same concentration as in plasma. The primary route of elimination for oseltamivir metabolites is urinary excretion, while a small amount of both metabolites (oseltamivir carboxylate and phosphate) can be found in faeces (Patel et al., 2015).

##### 2.3.1.2 Rationale for use in COVID-19

Oseltamivir seems effective in COVID-19 and currently is in different phases of clinical trials for COVID-19 (NCT04973462, NCT04558463). Although oseltamivir has been proven safe and well-tolerated in patients with CKD and ESKD (NCT01556633), these patients have been excluded from most clinical trials of oseltamivir against COVID-19. It may be due to, patients with CKD (3-4 stage) and ESKD, being more prone to infections like H1N1 or COVID-19 (Kute et al., 2011). Unlike other antivirals, oseltamivir possesses mild side effects however, it does not promote any life-threatening events (Choo et al., 2011). Moreover, ESKD patients can be treated with oseltamivir. Recently, results from a study on H1N1-infected ESKD patients show that treatment with 75 mg oseltamivir for 5 days had protective effects and was well tolerated in ESKD patients.

##### 2.3.1.3 Limitations and adverse effects

Patients with severe kidney impairment have been also excluded from recent clinical trials regarding oseltamivir against COVID-19 (NCT04338698, NCT04973462). Importantly, the pharmacokinetics of oseltamivir does not change with severe kidney impairment. Overall, a low dose of oseltamivir could prove a safe, tolerable, and effective treatment option for COVID-19 patients with CKD and ESKD.

Oseltamivir is safe in patients with hepatic and kidney dysfunction. Patients with a creatinine clearance <10 ml/min show high exposure to oseltamivir carboxylate; thus, a low dose of oseltamivir (75 mg/kg) is recommended in this group (Table 1) (Patel et al., 2015). However, the efficacy of low-dose oseltamivir on COVID-19 in patients with CKD is still unknown. On the other side, severe skin reactions can be seen in a patient treated with oseltamivir. Other adverse events include skin peeling, loosening of the skin, blistering, skin ulcers, and nose bleeding (Choo et al., 2011) (Table 1).

### 2.4 Nucleoside reverse transcriptase inhibitors (NRTIs)

#### 2.4.1 Azvudine

Azvudine is the first double-target nucleoside drug (inhibits nucleoside reverse transcriptase and restores expression of cytidine deaminase) having broad-spectrum antiviral activity against HIV, HCV, EV71, and HBV infections (Figure 1E) (Zhang et al., 2021). It is known to modulate the expression of P-glycoprotein (P-gp) and is also effective against SARS-CoV-2. Outcomes of a recent clinical trial (NCT04668235) showed that 40% of people treated with azvudine improved clinical symptoms (Zhang et al., 2021). Recently, the Chinese authorities approved Azvudine for the treatment of COVID-19. Azvudine has shown desirable pharmacokinetic properties, with excellent efficacy and safety, in its initial clinical trials (NCT04303598, CXHS2000016, CXHS2000017). During the clinical studies the azvudine 5 mg per day in combination with standard treatment for up to 14 days were given in COVID-19 patients (NCT05033145, NCT04668235).

##### 2.4.1.1 Rationale for use in COVID-19

The drug did not show kidney-related adverse events in COVID-19 patients (Ren et al., 2020). However, the exclusion of patients with GFR ≤60 ml/min/1.73 m2 from this study requires more information about this drug (Ren et al., 2020). In another study, kidney function parameters were found normal during treatment of azvudine. Oral administration of azvudine in rhesus macaques (SARS-CoV-2 infected) as well as in COVID-19 patients revealed that it reduces viral load, reduces inflammation, and organ damage (Zhang et al., 2021). Therefore, after checking the safety profile of this drug, the application was given priority in the review process against HIV patients by US FDA (CXHS2000016, CXHS2000017). Based on these reports, we speculate that azvudine might be repurposed for HIV-infected and CKD patients who are having COVID-19 infection. However, more clinical trials and a large sample size are required to further investigate this drug.

##### 2.4.1.2 Limitations and adverse effects

Data regarding the use of azvudine against COVID-19 patients with severe kidney impairment are limited, and do not report any adverse renal events. Moreover, but considering its excellent pharmacokinetics observed in different phases of clinical trials (GQ-FNC-2014–2, GQ-FNC-201, and NCT04109183) it might be the drug of potential against COVID-19 patients with CKD (Table 1). Azvudine shows mild and transient side effects which are less severe than other antivirals. Dizziness and nausea occurred in the approx. 10% of patients were treated with azvudine (Zhang et al., 2021).

### 2.5 HIV integrase inhibitors/non-nucleoside reverse transcriptase inhibitors (NNRTIs)

#### 2.5.1 Dolutegravir/rilpivirine

Dolutegravir is another integrase inhibitor approved in 2013 for the management of HIV (Figures 1H, 2A). Dolutegravir was approved by US-FDA against HIV infection and used for 4-week-old infants who never received an integrase strand inhibitor (DBCOND0129755, NCT04229290) (Ruel et al., 2022). It is combined with rilpivirine (non-nucleoside reverse transcriptase inhibitors) for HIV-infected patients whose viral load is more than <50 copies/ml. After administration dolutegravir binds to viral integrase (an enzyme that catalyzes the transfer of viral genetic material to human chromosomes. Recently, dolutegravir was effective against HIV in patients having COVID-19 (Gutierrez et al., 2021). The recommended daily dose is between 40–50 mg in HIV patients with or without COVID-19.

##### 2.5.1.1 Pharmacokinetic profile

Dolutegravir shows rapid administration and reaches *t*_max_ within 2 h (Ruel et al., 2022). A tight protein binding (>99%) and longer t_1/2_ (13–14 h in healthy and 11–12 h in HIV-infected patients) are the main characteristics of this drug. Therefore, its antiviral response lasts for 2–3 days after the last dose. Multiple daily doses were found to be more effective with a high C_trough_ 25 times greater than *in vitro* threshold. Dolutegravir is mainly metabolized by UGT1A1 and partially by CYP3A4 (Kobayashi et al., 2011). Dolutegravir inhibits CYP3A4 but is ineffective against other CYP enzymes (CYP-1A1, 2A6, 2B6, 2C8, and 2C9). It also inhibits the renal transporter OCT2 when C_max_ is 7.97–14.7 μM (Cottrell et al., 2013). Fatty meals can alter the pharmacokinetics of the dolutegravir. After absorption, it shows good tissue distribution and is seen in cerebrospinal fluid, male and female genital tract, and colorectal tissue and also crosses the blood-brain barrier. Less than <1% of the administered dose is excreted unchanged in the urine, which promises no dose adjustment required for patients with kidney impairment (Table 1) (Kreft et al., 2019).

##### 2.5.1.2 Rationale for use in COVID-19

Dolutegravir treatment suppresses viral load and does not affect kidney function. Moreover, dose adjustment is not required for dolutegravir in CKD patients, however, ESKD and patients on hemodialysis required continuous monitoring (NCT01353716). More importantly, dolutegravir’s pharmacokinetic profile indicates high plasma protein binding and low volume of distribution suggesting that it would not be affected by hemodialysis therefore can be given to patients who are on hemodialysis (Kreft et al., 2019). Moreover, it is safe to use in patients having viral load from 6 months with creatinine clearance rate less than <60 ml/min (CKD condition) (Kreft et al., 2019). In a retrospective study combination of dolutegravir/atazanavir/hydroxychloroquine treatment was found to be more effective against COVID-19 than a combination of lopinavir/ritonavir (Kalantari et al., 2021). Patients treated with dolutegravir/atazanavir/hydroxychloroquine showed minimal changes in creatinine level (initial-1.28 ± 0.90 during discharge-1.25 ± 0.72) but a change in urea levels (initial-22.54 ± 26.41, during discharge-30.02 ± 32.64) (Kalantari et al., 2021). This combination also showed higher activated partial thromboplastin time, decrease in C-reactive protein, potassium level, viral load, and cause less severe disease course. Therefore, by considering its safety concerns in patients with critical conditions (especially those with hemodialysis), dolutegravir might be a beneficial antiviral that can be used in CKD patients including patgients on hemodialysis.

##### 2.5.1.3 Limitations and adverse effects

Dolutegravir does not show any major or dose-dependent toxicity. Dolutegravir inhibits the OCT2 and multidrug and toxin extrusion transporter (MATE)1 therefore it may affect the level of drugs eliminated *via* OCT2 and MATE1 such as dofetilide and dalfampridine. This inhibition of OCT2 increases tubular uptake of creatinine and reduces creatinine clearance thus altering the plasma creatinine level. Therefore, increased plasma creatinine levels can be seen during the treatment with dolutegravir (Eron et al., 2013; Koteff et al., 2013). However, it might get misinterpreted with severe kidney-impaired patients treated with dolutegravir. Though dolutegravir increases plasma creatinine levels, no true kidney-related adverse events have been reported.

A report from a single-dose clinical trial evaluating dolutegravir pharmacokinetic properties revealed that dolutegravir shows 1% adverse events (dizziness and nasal congestion) compared to other drugs such as efavirenz (8%) (Ruel et al., 2022). Overall, besides the less evidence in patients with COVID-19, dolutegravir might prove beneficial against COVID-19 patients with severe kidney-related complications.

### 2.6 Nucleotide polymerase inhibitors

#### 2.6.1 Sofosbuvir

Sofosbuvir, a pyrimidine nucleotide derivative is an antiviral medication prescribed for the treatment of hepatitis C (HCV) (Figure 2B) (Akhil et al., 2018). Both, the FDA and the European Medicines Agency approved sofosbuvir and it was made commercially accessible in the US to treat chronic hepatitis C in 2013 (Pawlotsky, 2013). It is metabolized by the liver and converted into its active form 2′-α-fluoro-β-C-methyluridine-5′-triphosphate (Wiemer, 2020). It directly inhibits nonstructural 5B (NS5B) HCV RNA polymerase which is required for the synthesis and replication of HCV RNA (Rodríguez-TorresSofosbuvir (GS-, 2013). It is taken at a dose of 400 mg once daily and generally given in combination with ribavirin, and velpatasvir. Many clinical trials (Phase II/III/IV) have been conducted for evaluating the efficacy of sofosbuvir against COVID-19 (NCT04460443, NCT04498936, NCT04443725, NCT04535869, and NCT04530422). Sofosbuvir 400 mg with ravidasvir 200 mg or daclatasvir 60 mg, orally for 10 days are used in the treatment of COVID-19 (Abbass et al., 2021) (Zein et al., 2022).

##### 2.6.1.1 Pharmacokinetic profile

Sofosbuvir and its primary metabolite GS-331007, upon oral treatment, reach their peak plasma concentrations within 0.5-2 and 2–4 h, respectively without any impact of food (Kirby et al., 2013). It shows around 61–65% binding to human plasma proteins and is primarily cleared *via* kidneys (78%) in its metabolite form, GS-331007. Sofosbuvir and GS-331007 had average half-lives of 0.4 and 27 h, respectively (Rodríguez-TorresSofosbuvir (GS-, 2013). As per the reported evidence, epidemiological variables such as age, gender, race, or body mass index do not have a significant impact on the pharmacokinetics of sofosbuvir.

##### 2.6.1.2 Rationale for use in COVID-19

Sofosbuvir can be given to patients whose creatinine clearance is greater than 30 ml/min without adjusting the dose, however, it is not recommended for those patients having creatinine clearance less than 30 ml/min or receiving hemodialysis or half dose (200 mg) should be prescribed in such cases (Table 1) (Smolders et al., 2016; Taneja et al., 2018). As these recommendations are reported for HCV patients with CKD, so further investigations are required for evaluating the efficacy and tolerability of sofosbuvir in patients with COVID-19 along with CKD. In combination with ravidasvir or daclatasvir, it showed improvements in clinical symptoms, oxygen saturation, and decrease incidence of mortality in moderate to severe COVID-19 patients (Abbass et al., 2021; Zein et al., 2022).

##### 2.6.1.3 Limitations and adverse effects

Sofosbuvir-induced AKI has been observed in patients having moderate to severe kidney impairment. The condition can be reversed to normal on discontinuation of sofosbuvir therapy (Carrier et al., 2021). However, a retrospective analysis showed no change in the eGFR of CKD patients taking a sofosbuvir-based regimen (Sulkowski et al., 2022).

Also, sofosbuvir should not be given along with rifabutin and carbazepine as these medications possess strong P-glycoprotein inducing ability which can markedly reduce the plasma concentration of sofosbuvir and thereby, resulting in diminished therapeutic efficacy (Cholongitas and Papatheodoridis, 2014). Also, female patients and female spouses of male patients receiving any sofosbuvir-containing therapy should avoid pregnancy for up to 6 months after treatment ends (Rodríguez-TorresSofosbuvir (GS-, 2013).

### 2.7 Fusion inhibitors

#### 2.7.1 Umifenovir (arbidol)

Umifenovir is a drug made in Russia with antiviral properties on certain enveloped and non-enveloped viruses (Figure 2G). Umifenovir has been used against influenza A and B viruses, and hepatitis C virus (Chen et al., 2021). It inhibits the fusion of the virus lipid shell and cell membrane, thus preventing contact and penetration of the virus into host cells (Nojomi et al., 2020). Umifenovir can inhibit SARS-Cov-2 infection by interfering with the release of the virus from intracellular vesicles (Nojomi et al., 2020).

##### 2.7.1.1 Pharmacokinetic profile

The administration of umifenovir by oral route shows rapid absorption with an estimated *t*_max_ of 0.65–1.8 h (Deng et al., 2013). Umifenovir is quickly distributed in the stomach, intestines, and extremely in lungs. Its viral inhibition potency is found with EC_50_ at 4.11 μm. However, the high distribution of umifenovir in the lungs does not help to clear the viral load. Further, distributed umifenovir undergoes phase I and phase II metabolism in humans (Chen et al., 2021). The P450s and FMOs to umifenovir equally contributed to the metabolism of umifenovir. FMOs carry out sulfoxidation while P450s produce different metabolites of umifenovir such as M5, M6-1, M7, and M8. Wherein, M6-1 is considered the most responsible metabolite having a long elimination half-life (∼25h). Because of phase I and phase II metabolism, the *t*_1/2_ of umifenovir is less than its metabolites. Umifenovir is mainly excreted in faeces, nearly 40% of the administered dose is excreted unchanged, of which approximately 38.9% is excreted in the bile and 0.12% through the kidneys (Deng et al., 2013). Moreover, 31 metabolites were found in urine, 16 in plasma, and 24 in faeces when 200 mg umifenovir was administered. Whereas phase II sulfate and glucuronide conjugates are mainly found in urine and the primary parent drug in the faeces (Deng et al., 2013).

##### 2.7.1.2 Rationale for use in COVID-19

It has high lung distribution and hence can be a better therapeutic option against COVID-19 (NCT04350684). It has been studied in combination with other antivirals (NCT04350684). However, in CKD and immunocompromised patients, the use of umifenovir is not suitable (Lian et al., 2020). A phase III trial suggested that umifenovir was efficacious in mild-asymptomatic COVID-19 patients when given at the dose of 800 mg twice a day for a maximum of 14 days (Ramachandran et al., 2022). However, a multicenter retrospective revealed that umifenovir treatment is associated with increased in-hospital mortality (Zhou et al., 2021). The non-survival group had a higher incidence of renal insufficiency (14%) than the survival group. Moreover, out of 109 patients, almost 28 patients show acute kidney injury (Zhou et al., 2021). This suggests that umifenovir is not a good choice for chronic administration in COVID-19 patients having CKD (Table 1).

##### 2.7.1.3 Limitations and adverse effects

Umifenovir shows digestive symptoms like nausea and vomiting. Mainly reported for causing acute kidney injury upon chronic administration in COVID-19 patients having later stages of CKD or ESKD. Not reported for any severe systemic or liver toxicities.

## 3 Monoclonal antibodies for the treatment of COVID-19

Few monoclonal antibodies (mAb) have received EUAs from FDA to be used in COVID-19 patients having CKD and other severe diseases that require hospitalization.

### 3.1 Bamlanivimab/etesevimab (BEC)

This combination is preferred for mild to moderate COVID-19. According to cohort study, early administration of BEC should be encouraged in COVID-19 patients with kidney impairment. The dose of 700 mg of bamlanivimab and 1400 mg of etesevimab can be given to renal-impaired patients without dose adjustment (Vena et al., 2021). In solid organ transplant (SOT) patients having mild to moderate COVID-19 symptoms, this combination was found well-tolerated however it was not potential enough in preventing hospitalization (Kutzler et al., 2021). The early administration of this combination reduced the rate of hospitalization and external oxygen supply on COVID-19 patients.

### 3.2 Casirivimab/imdevimab

This combination received EUA to treat non-hospitalized patients. Both of these drugs are not eliminated through the urine or metabolized by P450 therefore, this combination would be safe for COVID-19 patients, especially those who are having CKD or are on dialysis treatment (Liu et al., 2021). One case report suggests that no dose adjustment is required for these drugs for kidney-impaired patients. Moreover, due to their high molecular weight, this combination can be administered before, during, or after hemodialysis (Terakawa et al., 2022). According to one study, the treatment of casirivimab/imdevimab in solid organ transplant patients having COVID-19 didn’t experienced the progression of symptoms or required hospitalization (Dhand et al., 2021). Among COVID-19 patients the single treatment of combination of 700 mg of bamlanivimab and 1400 mg, of etesevimab i.v., significantly reduced the viral load, hospitalizations and all-cause mortality.

### 3.3 Sotrovimab and tocilizumab

For these mAbs no dose adjustment is required in COVID-19 patients having CKD or treated with dialysis. Moreover, these drugs are found to be safe and effective even in patients who are on chronic hemodialysis and those who underwent solid-organ transplantation (Mambelli et al., 2022). Sotrovimab infusion (500 mg) is found to be well tolerated in organ transplant patients having COVID-19. Similarly, tocilizumab (8 mg/kg, twice) is able to decrease the cytokine levels in kidney-transplanted patients having COVID-19 (Pérez-Sáez et al., 2020).

### 3.4 Baricitinib

Baricitinib reduces inflammatory cascades and immune system activation by inhibiting Janus kinase. Baricitinib received EUA in combination with remdesivir as COVID-19 treatment (Kelton et al., 2022). The dose of 2–4 mg/day for 14 days with remdesivir was given. Almost 75% of the drug is eliminated by kidneys resulting in more adverse effects when kidney function declines. Dose modification is recommended in people whose eGFR is below 60 ml/min. Baricitinib is not indicated for AKI or dialysis patients.

### 3.5 Adalimumab and sarilumab

Adalimumab and sarilumab targets tumor necrosis factor (TNF- α) and IL-6 receptor respectively in COVID-19 (Patel and Wadhwa, 2021). Adalimumab (40 mg along with SoC) has been demonstrated to be safe in CKD and dialysis patients without any impairment of renal function and can be given without dose modification (Abdalla et al., 2020). Similarly, sarilumab (200 mg, i.v. twice or 400 mg i.v. single dose) can be given to patients with mild to moderate renal failure without any dose modification (Chamlagain et al., 2021). It is not eliminated by kidneys as the breakdown of sarilumab into peptides, and amino acids occur *via* catabolic pathways instead of kidney or hepatic mechanisms. However, its usage in patients with severe renal impairment has not been evaluated.

## 4 Discussion

Even after the approval of few vaccines and drugs to combat COVID-19, the associated morbidity and mortality rate are not under control worldwide. The reason is the emergence of new coronavirus variants with persistent change in their genome sequences (https://www.cdc.gov/coronavirus/2019-ncov/variants/variant-classifications). Vaccines are not totally effective in case of variant of high consequence, wherein the further booster doses are necessary (Meng et al., 2022). Also, there are few reports suggesting the adverse events after vaccination which require further clinical trials on large populations for longer period of time (Fraiman et al., 2022; Garg and Paliwal, 2022; Patone et al., 2022). However, conducting clinical trials for evaluating the clinical efficacy and safety of vaccines, and drugs like antivirals, steroids, or mAbs is costly and lengthy. Moreover, inclusion of comorbid patients for screening and evaluation of the confirmatory effects of such therapies is not possible in a small populations and/or in a very short time. Unfortunately, many of the clinical trials excluded patients with diabetes, cardiovascular, or kidney disease. Hence, more clinical trials with larger comorbid populations would need to be conducted to address all aforementioned issues.

**FIGURE 3:**
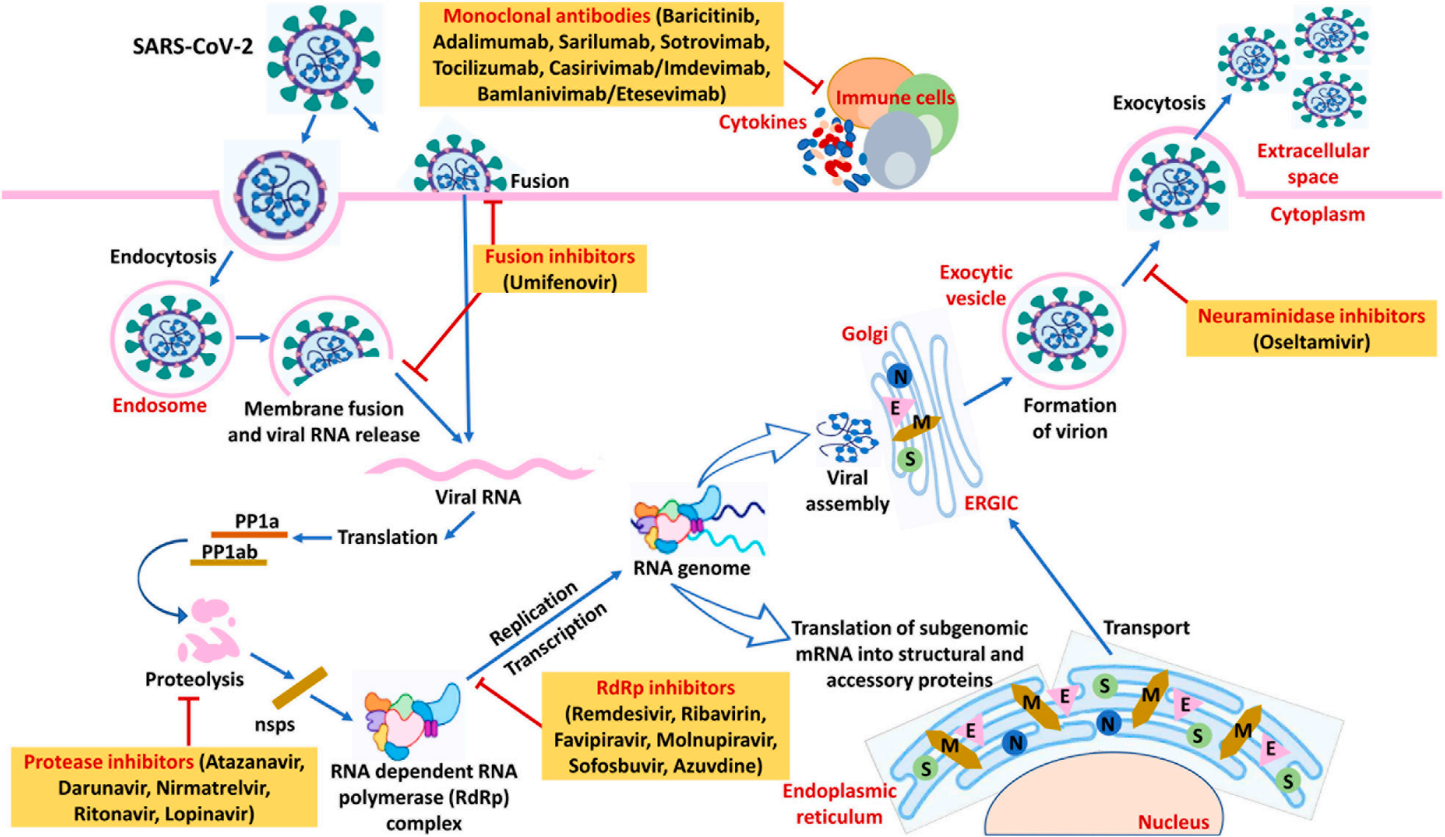
An overview of SARS-CoV-2’s life cycle along with the potential targets of anti-viral drugs and monoclonal antibodies in COVID-19. On host cells, the spike protein (S) of SARS-CoV-2 interacts with the cellular receptor ACE2 followed by viral entry into the host cell. It happens either by fusion of the virus with plasma membrane due to activation by serine protease or activation by endocytic machinery of the host resulting in viral and cellular membranes fusion. The viral genome is unveiled in the cytoplasm and translated to viral replicase polyproteins (PP1a and PP1ab) followed by subsequent cleavage to form nonstructural proteins (nsps) *via* proteinases of the virus. Some of the nsps consequently form the replicase-transcriptase complex as RNA-dependent RNA polymerase (RdRp). Through intermittent transcription, the polymerase generates subgenomic mRNA series that is ultimately translated into functional viral proteins. In the cytoplasm, viral nucleocapsids are formed with genomic RNA and N proteins accompanied by budding in the ERGIC lumen. The virus is then released from the host’s infected cell to the extracellular space *via* exocytosis. Medications with significant anti-SARS-CoV-2 action including anti-viral drugs and monoclonal antibodies acting on the different phases of the virus’s life cycle have been also illustrated in the figure. Abbreviation- S: Structural proteins, E: Envelope, M: Membrane, N: Nucleocapsid, ACE2: Angiotensin-converting enzyme 2, ERGIC: Endoplasmic Reticulum- Golgi intermediate compartment.

From the published evidence, it is well proven that in CKD patients with COVID-19 the rates of hospitalization and death are higher (Mirijello et al., 2021; Goh et al., 2022). These patients are more prone to adverse drug effects due to drug-drug reactions or altered pharmacokinetics and pharmacodynamics because of kidney dysfunction. CKD implies an impaired clearance of many antiviral drugs (Steiger et al., 2022). Hence, a careful choice of antiviral drugs for patients with COVID-19 having CKD is a challenging task for clinicians. Not all broad-spectrum antivirals recommended for COVID-19 patients are safe in COVID-19 patients with CKD. For example, remdesivir shall not be prescribed in COVID-19 patients having CKD stage G3-G5 or ESKD. Because, remdesivir is eliminated *via* the kidneys wherein in case of decreased eGFR its carrier sulfobutylether-β-cyclodextrin accumulates in tubules and cause nephrotoxicity (Luke et al., 2012). Similarly, chronic use of ATV is not recommended as it may cause interstitial nephritis and glomerulosclerosis in patients with kidney disease. Molnupiravir, oseltamivir, azvudine, famciclovir, and dolutegravir are relatively safe and do not require dose adjustments or cause nephrotoxicity in COVID-19 patients having CKD. However, molnupiravir is not recommended for the patients of less than 18 years’ age, pregnant women, and elderly people. Sofosbuvir requires special precautions and close monitoring. Sofosbuvir should not be combined with rifabutin and carbazepine. Furthermore, it has the potential to cause AKI in patients with moderate to severe CKD. Female patients and female spouses of male patients receiving any sofosbuvir-containing therapy should avoid pregnancy for up to 6 months after the end of treatment. The use of favipiravir and nirmatrelvir/ritonavir combination should be avoided in patients with kidney (and liver) dysfunction as it can worsen clinical outcomes in such patients. CKD patients with a GFR >60 ml/min/1.73 m^2^ can be given the recommended dose. Ribavirin requires monitoring of hematological parameters for hemolytic anaemia. Additionally, it is not recommended for pregnant and breastfeeding women due to its teratogenic effects. Ribavirin is to be avoided in CKD patients with a GFR <90 ml/min/1.73 m^2^. Umifenovir and lopinavir/ritonavir combination shall be avoided in COVID-19 patients with CKD. The use of mAbs in mild to moderate COVID-19 patients are recommended. However, special attention is required when administering to the severe or hospitalized patients. In the kidney impaired and SOT patients the combinations of amlanivimab/etesevimab, casirivimab/imdevimab, sotrovimab and tocilizumab are safe and can be preferred over antivirals and other mAbs. However, baricitinib mAbs shall not be given to the patients with low eGFR and who underwent for SOT. The adalimumab and sarilumab mAbs can be used in CKD and dialysis patients. An overview of SARS-CoV-2’s life cycle along with the potential targets of anti-viral drugs and monoclonal antibodies in COVID-19 is described in Figure 3. These findings are clearly indicating that while selecting the dose, route and frequency of drugs the presence of CKD or its stages must be taken into consideration. The summary of the dose, frequency, clinical indications and efficacy of these antivirals and mAbs can be seen in Table 2.

**TABLE 2:** Dosage, clinical indications and efficacy of antivirals and monoclonal antibodies in COVID-19 patients.

Class/drugs	Dosage/route/duration and clinical indications	Patients characteristics/recommendations	Clinical efficacy in COVID-19
RNA-dependent RNA polymerase (RdRp) inhibitor			
Remdesivir	Mild-to-moderate COVID-19 patients: 200 mg i.v. on the 1st day, 100 mg for the next 2–3 days	Mild-to-moderate COVID-19 patients	Shortens the time of recovery from COVID-19
	Severe COVID-19: above treatment can be extended up to 10 days	Hospitalized and severe COVID-19 patients	Lowers respiratory tract-related complications
	In adults with eGFR ≥60: 4 mg/kg, orally/day	In COVID-19 patients with eGFR ≥60 ml/min/1.73 m2	
	In pediatric patients, 5 mg/kg on day 1 followed by 2.5 mg/kg		
Molnupiravir	Moderate to severe COVID-19: 800 mg/oral/12 h for 5 days	Moderate to severe COVID-19 patients	Lowers the risk of hospitalization
		COVID-19 patients with mild-to-moderate kidney dysfunction	Decreased the % death among unvaccinated adults
		Patients with influenza and coronavirus infections	
Famciclovir	Dose ranges from 125 to 1500 mg/day (depending on the status of kidney function in COVID-19 patients)	COVID-19-associated pneumonia	Inhibits cell-mediated inflammation and immune cells activations
		COVID-19 patients with kidney impairment or hemodialysis (with low dose)	Accelerates viral clearance and prevents swelling and vesiculation
		Patients with herpes simplex virus infections (varicella zoster and herpes zoster)	Gradually improves kidney functions
Ribavirin	COVID-19: 400 mg/twice/daily when taken with lopinavir/ritonavir or 500 mg twice/thrice/day	Mild-to-moderate COVID-19 patients without kidney impairment	Shortens the duration of hospitalizations
	Hepatitis-C: 800–1200 mg/day	Hepatitis-C patients	Increases the efficacy of lopinavir/ritonavir
Favipiravir	COVID-19: maintenance dose of 200–600 mg twice daily and various loading doses of 1600, 1800, and 2400 mg for 10 days	COVID-19	No promising results in COVID-19 patients with or without kidney dysfunction
	Influenza: 1600 mg twice daily on day1 and 600 mg twice daily from day2–5	Influenza	
Protease inhibitors			
Atazanavir	COVID-19: 300–400 mg/day/oral along with other antivirals	COVID-19 and COVID-19 with moderate kidney impairment or receiving dialysis	Improves oxygen saturation, clinical and paraclinical characteristics of COVID-19
	HIV infection: 400 mg/oral/day or 300 plus 100 mg of ritonavir/oral/day	HIV.	
Darunavir	COVID-19: 800 mg with 150 mg of cobicistat per day for 5–7 days along with conventional therapies	COVID-19 and COVID-19 with kidney impairment	Controversial outcomes regarding the efficacy and safety
Nirmatrelvir/ritonavir combination	COVID-19: a) Patients having eGFR ≥60 ml/min: 300 mg of nirmatrelvir and 100 mg of ritonavir twice daily for 5 days	COVID-19 and COVID-19 with kidney impaired patients having eGFR ≥30 ml/min	Significantly lowered the mortality rate. Reduces the risk of hospitalization or progression to severe COVID-19 infection in vaccinated patients
	b) Patients having an eGFR ≥30–60 ml/min: 150 mg og nirmatrelvir and 100 mg of ritonavir twice daily for 5 days		
Lopinavir/ritonavir combination	COVID-19: 400 mg (lopinavir) and 100 mg (ritonavir) per day/oral for 10 days	COVID-19 and COVID-19 with eGFR >90 ml/min/1.73 m^2^ as acute therapy	No confident data is available regarding the clinical efficacy
		Chronic hepatitis B or C	
Neuraminidase inhibitors			
Oseltamivir	COVID-19: 75 mg once or twice a day for 5–14 days, alone or in combination with drugs like HCQS or azithromycin	COVID-19 and COVID-19 with CKD or ESKD patients wherein TDM is required	Shorten hospital stays and reduced the mortality rate among hospitalized COVID-19 patients
		Influenza A and influenza B viruses	
Nucleoside reverse transcriptase inhibitors (NRTIs)			
Azvudine	COVID-19: 5 mg/oral/day in combination with standard treatment for up to 14 days	COVID-19 and COVID-19 with HIV or CKD patients	Improved COVID-19 related clinical symptoms
		HIV, HCV, EV71, and HBV infections	Reduces viral load, inflammation, and organ damage
HIV integrase inhibitors/non-nucleoside reverse transcriptase inhibitors (NNRTIs)			
Dolutegravir/Rilpivirine	In HIV patients with or without COVID-19: 40–50 mg/day up to 10 days	HIV patients with or without COVID-19	Higher activated partial thromboplastin time, and lower C-reactive protein and potassium level
		COVID-19 with CKD, ESKD or dialysis wherein TDM is required	Suppressed viral load and cause less severe disease course
Nucleotide polymerase inhibitors			
Sofosbuvir	COVID-19: Sofosbuvir 400 mg with ravidasvir 200 mg or daclatasvir 60 mg, orally for 7–10 days	COVID-19.Hepatitis-C	Improved clinical symptoms, oxygen saturation, and decreased incidence of mortality in moderate to severe COVID-19 patients
	Hepatitis-C: 400 mg/day and generally given in combination with ribavirin, and velpatasvir		
Fusion inhibitors			
Umifenovir (Arbidol)	COVID-19: 800 mg twice a day for a maximum of 14 days	COVID-19 (Mild-asymptomatic).Influenza A and B	Mild-asymptomatic COVID-19 patients were found clinically recovered and RT-PCR negative
Monoclonal antibodies			
Bamlanivimab/Etesevimab (BEC)	COVID-19: 700 mg of bamlanivimab and 1400 mg of etesevimab	COVID-19 with or without kidney impairment	Lower rate of hospitalization and reduced need for any supplementary oxygen
Casirivimab/Imdevimab	COVID-19: Casirivimab 2400 mg and imdevimab 1200 mg, single intravenous treatment	COVID-19 with or without CKD and dialysis	Significant reduction in viral load, hospitalizations and all-cause mortality
Sotrovimab and Tocilizumab	COVID-19: Sotrovimab (500 mg) and tocilizumab (8 mg/kg)	COVID-19 and kidney transplant recipient	Reduction in cytokines level, inflammation and all over clinical symptoms
Baricitinib	COVID-19: 2–4 mg/day for 14 days	COVID-19	Decreased immune cells activation and mortality rate
Adalimumab and Sarilumab	COVID-19: Adalimumab- 40 mg with SoC. Sarilumab- single 400 mg, i.v. or 200 mg in divided dose	COVID-19 with or without CKD and mild-moderate kidney failure	Decreased cytokine synthesis, inflammation and improvement in clinical symptoms and all-cause mortality rate

Abbreviations* COVID-19: Coronavirus Disease-2019, CKD: chronic kidney disease, ESKD: end stage kidney disease, HCQS: hydroxychloroquine, i.v.: Intravenous GFR: glomerular filtration rate, TDM: therapeutic drug monitoring, HIV: human immunodeficiency virus, HCV: hepatitis C virus, EV71: enterovirus 17, HBV: hepatitis B virus, SoC: standard of care, RT-PCR: reverse transcription-polymerase chain reaction test.

The current therapeutic strategies are based on either the reports from case studies or pharmacokinetic and pharmacodynamics of the drugs. However, further determination of the safe and effective dose, its frequency, and evaluation of the clinical efficacy and safety of these antivirals and mAbs alone or in different combinations need to be studied in large population. This review may help to select the appropriate drugs or combinations to treat the COVID-19 patients having different stages of CKD, and ESKD. It will also help and motivate the researchers to extend the preclinical and clinical research on antivirals in COVID-19 patients having CKD conditions.

## 5 Conclusion

The order (safest to least safe) of antivirals based on their safety profile in COVID-19 infected CKD patients is as follows. Among the approved antivirals: Azvudine, Molnupiravir (Lagevrio), Nirmatrelvir with Ritonavir (Paxlovid), Remdesivir (Veklury) and among the non-approved or those are under the clinical pipeline: Oseltamivir, Famciclovir, Dolutegravir/Rilpivirine, Darunavir, Atazanavir, Ribavirin, Favipiravir, Nirmatrelvir/ritonavir combination, Sofosbuvir, Umifenovir, Lopinavir/ritonavir combination. We recommend not to use the antivirals like Sofosbuvir, Umifenovir, Favipiravir, and Lopinavir/Ritonavir combination in CKD patients. The combinations of mAbs such as amlanivimab/etesevimab, casirivimab/imdevimab, sotrovimab and tocilizumab can be used in CKD, kidney impaired and SOT patients over the other mAbs and non-safe antivirals.

These suggestions are made based on the pharmacokinetics and pharmacodynamics profile of the respective antivirals and mAbs, and outcomes from the observational and interventional clinical studies.

## References

[B1] Abbass S. Kamal E. Salama M. Salman T. Sabry A. Abdel-Razek W. (2021). Efficacy and safety of sofosbuvir plus daclatasvir or ravidasvir in patients with COVID-19: A randomized controlled trial. J. Med. Virology 93 (12), 6750–6759. 10.1002/jmv.27264 34379337 PMC8426808

[B2] Abdalla S. Elgassim L. Rustom F. Othman M. (2020). Acute kidney injury caused by darunavir in a patient with COVID-19: A case report. Open J. Nephrol. 10 (4), 375–382. 10.4236/ojneph.2020.104037

[B3] Akhil M. S. Kirushnan B. Martin M. Arumugam K. Ganesh Prasad N. K. Ravichandran R. (2018). Sofosbuvir‐based treatment is safe and effective in Indian hepatitis C patients on maintenance haemodialysis: A retrospective study. Nephrology 23 (5), 446–452. 10.1111/nep.13050 28339162

[B4] Bausch D. G. Hadi C. M. Khan S. H. Lertora J. J. L. (2010). Review of the literature and proposed guidelines for the use of oral ribavirin as postexposure prophylaxis for Lassa fever. Clin. Infect. Dis. 51 (12), 1435–1441. 10.1086/657315 21058912 PMC7107935

[B5] Beigel J. H. Tomashek K. M. Dodd L. E. Mehta A. K. Zingman B. S. Kalil A. C. (2020). Remdesivir for the treatment of covid-19 — final report. N. Engl. J. Med. 383 (19), 1813–1826. 10.1056/NEJMoa2007764 32445440 PMC7262788

[B6] Best B. M. Diep H. Rossi S. S. Farrell M. Williams E. Fau - Lee G. (2011). Pharmacokinetics of lopinavir/ritonavir crushed versus whole tablets in children. J. Acquir Immune Defic. Syndr. 58 (4), 385–391. 10.1097/QAI.0b013e318232b057 21876444 PMC3205189

[B7] Binois Y. Hachad H. Salem J. E. Charpentier J. Lebrun-Vignes B. Pène F. (2020). Acute kidney injury associated with lopinavir/ritonavir combined therapy in patients with COVID-19. Kidney Int. Rep. 5 (10), 1787–1790. 10.1016/j.ekir.2020.07.035 32838087 PMC7409925

[B8] Bosaeed M. Alharbi A. Mahmoud E. Alrehily S. Bahlaq M. Gaifer Z. (2022). Efficacy of favipiravir in adults with mild COVID-19: A randomized, double-blind, multicentre, placebo-controlled clinical trial. Clin. Microbiol. Infect. 28 (4), 602–608. 10.1016/j.cmi.2021.12.026 35026375 PMC8747778

[B9] Brown D. G. Wobst H. J. (2021). A decade of FDA-approved drugs (2010–2019): Trends and future directions. J. Med. Chem. 64 (5), 2312–2338. 10.1021/acs.jmedchem.0c01516 33617254

[B10] Brown P. A. McGuinty M. Argyropoulos C. Clark E. G. Colantonio D. Giguere P. (2022). Early experience with modified dose nirmatrelvir/ritonavir in dialysis patients with coronavirus disease-2019. medRxiv. 2022:2022.05.18.22275234.

[B11] Busti A. J. Hall R. G. Margolis D. M. (2004). Atazanavir for the treatment of human immunodeficiency virus infection. Pharmacother. J. Hum. Pharmacol. Drug Ther. 24 (12), 1732–1747. 10.1592/phco.24.17.1732.52347 15585441

[B12] Cao Z. Gao W. Bao H. Feng H. Mei S. Chen P. (2022). VV116 versus nirmatrelvir–ritonavir for oral treatment of covid-19. N. Engl. J. Med. 10.1056/nejmoa2208822 PMC9812289 36577095

[B13] Capone V. Cuomo V. Esposito R. Canonico M. E. Ilardi F. Prastaro M. (2020). Epidemiology, prognosis, and clinical manifestation of cardiovascular disease in COVID-19. Expert Rev. Cardiovasc. Ther. 18 (8), 531–539. 10.1080/14779072.2020.1797491 32672482

[B14] Carrier P. Debette-Gratien M. Bettayeb M. Essig M. Loustaud-Ratti V. (2021). Sofosbuvir and the risk of kidney dysfunction. J. Hepatology 74 (1), 256–257. 10.1016/j.jhep.2020.08.019 33071010

[B15] Castillo-Mancilla J. R. Aquilante C. L. Wempe M. F. Smeaton L. M. Firnhaber C. LaRosa A. M. (2016). Pharmacogenetics of unboosted atazanavir in HIV-infected individuals in resource-limited settings: A sub-study of the AIDS clinical trials group (ACTG) PEARLS study (NWCS 342). J. Antimicrob. Chemother. 71 (6), 1609–1618. 10.1093/jac/dkw005 26892777 PMC4867099

[B16] Chamlagain R. Shah S. Sharma Paudel B. Dhital R. Kandel B. (2021). Efficacy and safety of sarilumab in COVID-19: A systematic review. Interdiscip. Perspect. Infect. Dis. 2021, 8903435. 10.1155/2021/8903435 34721573 PMC8556127

[B17] Chen C. Zhang Y. Huang J. Yin P. Cheng Z. Wu J. (2021). Favipiravir versus arbidol for clinical recovery rate in moderate and severe adult COVID-19 patients: A prospective, multicenter, open-label, randomized controlled clinical trial. Front. Pharmacol. 12, 683296. 10.3389/fphar.2021.683296 34539392 PMC8443786

[B18] Chen C. Zhang Y. Huang J. Yin P. Cheng Z. Wu J. (2020). Favipiravir versus arbidol for COVID-19: A randomized clinical trial. MedRxiv. 10.3389/fphar.2021.683296 PMC8443786 34539392

[B19] Choi J. Horner K. A. Carnevale K. (2020). Atazanavir. StatPearls [internet]. Treasure Island, FL: StatPearls Publishing. 31869072

[B20] Cholongitas E. Papatheodoridis G. V. (2014). Sofosbuvir: A novel oral agent for chronic hepatitis C. Ann. Gastroenterology Q. Publ. Hellenic Soc. Gastroenterology 27 (4), 331–337. PMC4188929 25332066

[B21] Choo D. Hossain M. Liew P. Chowdhury S. Tan J. (2011). Side effects of oseltamivir in end-stage renal failure patients. Nephrol. Dial. Transplant. 26 (7), 2339–2344. 10.1093/ndt/gfq737 21193643

[B22] Cottrell M. L. Hadzic T. Fau - Kashuba A. D. M. Kashuba A. D. (2013). Clinical pharmacokinetic, pharmacodynamic and drug-interaction profile of the integrase inhibitor dolutegravir. Clin. Pharmacokinet. 52 (11), 981–994. 10.1007/s40262-013-0093-2 23824675 PMC3805712

[B23] De Clercq E. Li G. (2016). Approved antiviral drugs over the past 50 years. Clin. Microbiol. Rev. 29 (3), 695–747. 10.1128/CMR.00102-15 27281742 PMC4978613

[B24] Deb S. A-O. Reeves A. A. Hopefl R. Bejusca R. (2021). ADME and pharmacokinetic properties of remdesivir: Its drug interaction potential. Pharm. (Basel) 14 (7), 655. 10.3390/ph14070655 PMC8308800 34358081

[B25] Deng P. Zhong D Fau - Yu K. Yu K. Fau - Zhang Y. Zhang Y. Fau - Wang T. (2013). Pharmacokinetics, metabolism, and excretion of the antiviral drug arbidol in humans. Antimicrob. Agents Chemother. 57 (4), 1743–1755. 10.1128/AAC.02282-12 23357765 PMC3623363

[B26] Dhand A. Lobo S. A. Wolfe K. Feola N. Lee L. Nog R. (2021). Casirivimab-imdevimab for treatment of COVID-19 in solid organ transplant recipients: An early experience. Transplantation 105 (7), e68–e69. 10.1097/TP.0000000000003737 33724242

[B27] Dhawan M. Choudhary P. Choudhary O. (2022). Emergence of Omicron sub-variant BA.2: Is it a matter of concern amid the COVID-19 pandemic? Int. J. Surg. Lond. Engl. 99, 106581. 10.1016/j.ijsu.2022.106581 PMC8860472 35202859

[B28] Drożdżal S. Rosik J. Lechowicz K. Machaj F. Szostak B. Przybyciński J. (2021). An update on drugs with therapeutic potential for SARS-CoV-2 (COVID-19) treatment. Drug resistance updates: Reviews and commentaries in antimicrobial and anticancer chemotherapy. Drug Resist Updat 59, 100719. 10.1016/j.drup.2020.100719 PMC8654464 34991982

[B29] Du Y. X. Chen X. P. (2020). Favipiravir: Pharmacokinetics and concerns about clinical trials for 2019‐nCoV infection. Clin. Pharmacol. Ther. 108 (2), 242–247. 10.1002/cpt.1844 32246834

[B30] Duong B. V. Larpruenrudee P. Fang T. Hossain S. I. Saha S. C. Gu Y. (2022). Is the SARS CoV-2 omicron variant deadlier and more transmissible than delta variant? Int. J. Environ. Res. Public Health 19 (8), 4586. 10.3390/ijerph19084586 35457468 PMC9032753

[B31] Eron J. J. Clotet B. Fau - Durant J. Durant J. Fau - Katlama C. Kumar P. (2013). Safety and efficacy of dolutegravir in treatment-experienced subjects with raltegravir-resistant HIV type 1 infection: 24-week results of the VIKING study. J. Infect. Dis. 207 (5), 740–748. 10.1093/infdis/jis750 23225901 PMC3563307

[B32] Fischer W. Eron J. J. Holman W. Cohen M. S. Fang L. Szewczyk L. J. (2021). Molnupiravir, an oral antiviral treatment for COVID-19. MedRxiv.

[B33] Food U. S. Drug A. (2022). Fact sheet for healthcare providers: Emergency use authorization for molnupiravir.

[B34] Fraiman J. Erviti J. Jones M. Greenland S. Whelan P. Kaplan R. M. (2022). Serious adverse events of special interest following mRNA COVID-19 vaccination in randomized trials in adults. Vaccine 40 (40), 5798–5805. 10.1016/j.vaccine.2022.08.036 36055877 PMC9428332

[B35] Furuta Y. Gowen B. B. Takahashi K. Shiraki K. Smee D. F. Barnard D. L. (2013). Favipiravir (T-705), a novel viral RNA polymerase inhibitor. Antivir. Res. 100 (2), 446–454. 10.1016/j.antiviral.2013.09.015 24084488 PMC3880838

[B36] Garg R. K. Paliwal V. K. (2022). Spectrum of neurological complications following COVID-19 vaccination. Neurol. Sci. 43 (1), 3–40. 10.1007/s10072-021-05662-9 34719776 PMC8557950

[B37] Giampaoli O. Sciubba F. Biliotti E. Spagnoli M. Calvani R. Tomassini A. (2022). Precision medicine: Determination of ribavirin urinary metabolites in relation to drug adverse effects in HCV patients. Int. J. Mol. Sci. 23 (17), 10043. 10.3390/ijms231710043 36077436 PMC9456413

[B38] Gill K. S. Wood M. J. (1996). The clinical pharmacokinetics of famciclovir. Clin. Pharmacokinet. 31 (1), 1–8. 10.2165/00003088-199631010-00001 8827396

[B39] Goh B. L. Shanmuganathan M. Peariasamy K. Misnan N. A. Chidambaram S. K. Wong E. F. S. (2022). COVID-19 death and kidney disease in a multiracial Asian country. Nephrology 27 (7), 566–576. 10.1111/nep.14045 35438223 PMC9115296

[B40] Grein J. Ohmagari N. Shin D. Diaz G. Asperges E. Castagna A. (2020). Compassionate use of remdesivir for patients with severe covid-19. N. Engl. J. Med. 382 (24), 2327–2336. 10.1056/NEJMoa2007016 32275812 PMC7169476

[B41] Gutierrez M. D. M. Mur I. A-O. Mateo M. G. Vidal F. A-O. Domingo P. A-O. (2021). Pharmacological considerations for the treatment of COVID-19 in people living with HIV (PLWH). Expert Opin. Pharmacother. 22 (9), 1127–1141. 10.1080/14656566.2021.1887140 33634724 PMC7919104

[B42] Gutierrez‐Valencia A. Trujillo‐Rodriguez M. Fernandez‐Magdaleno T. Espinosa N. Viciana P. López‐Cortés L. F. (2018). Darunavir/cobicistat showing similar effectiveness as darunavir/ritonavir monotherapy despite lower trough concentrations. J. Int. AIDS Soc. 21 (2), e25072. 10.1002/jia2.25072 29430854 PMC5808101

[B43] Hafner V. Czock D. Burhenne J. Riedel K-D. Riedel K. Bommer J. (2010). Pharmacokinetics of sulfobutylether-beta-cyclodextrin and voriconazole in patients with end-stage renal failure during treatment with two hemodialysis systems and hemodiafiltration. Antimicrob. Agents Chemother. 54 (6), 2596–2602. 10.1128/AAC.01540-09 20368400 PMC2876385

[B44] Hammond J. Leister-Tebbe H. Gardner A. Abreu P. Bao W. Wisemandle W. (2022). Oral nirmatrelvir for high-risk, nonhospitalized adults with covid-19. N. Engl. J. Med. 386 (15), 1397–1408. 10.1056/NEJMoa2118542 35172054 PMC8908851

[B45] Hammond J. Leister-Tebbe H. Gardner A. Abreu P. Bao W. Wisemandle W. (2022). Oral nirmatrelvir for high-risk, nonhospitalized adults with covid-19. N. Engl. J. Med. 386 (15), 1397–1408. 10.1056/NEJMoa2118542 35172054 PMC8908851

[B46] Henry B. M. Lippi G. (2020). Chronic kidney disease is associated with severe coronavirus disease 2019 (COVID-19) infection. Int. urology Nephrol. 52 (6), 1193–1194. 10.1007/s11255-020-02451-9 PMC7103107 32222883

[B47] Hiremath S. McGuinty M. Argyropoulos C. Brimble K. S. Brown P. A. Chagla Z. (2022). Prescribing nirmatrelvir/ritonavir for COVID-19 in advanced CKD. Clin. J. Am. Soc. Nephrol. 17, 1247–1250. 10.2215/cjn.05270522 35680135 PMC9435977

[B48] Horby P. W. Mafham M. Bell J. L. Linsell L. Staplin N. Emberson J. (2020). Lopinavir–ritonavir in patients admitted to hospital with COVID-19 (RECOVERY): A randomised, controlled, open-label, platform trial. Lancet 396 (10259), 1345–1352. 10.1016/S0140-6736(20)32013- 33031764 PMC7535623

[B49] Humeniuk R. Mathias A. Cao H. Osinusi A. Shen G. Chng E. (2020). Safety, tolerability, and pharmacokinetics of remdesivir, an antiviral for treatment of COVID-19, in healthy subjects. Clin. Transl. Sci. 13 (5), 896–906. 10.1111/cts.12840 32589775 PMC7361781

[B50] Humeniuk R. Mathias A. Kirby B. J. Lutz J. D. Cao H. Osinusi A. (2021). Pharmacokinetic, pharmacodynamic, and drug-interaction profile of remdesivir, a SARS-CoV-2 replication inhibitor. Clin. Pharmacokinet. 60 (5), 569–583. 10.1007/s40262-021-00984-5 33782830 PMC8007387

[B51] Hung I. F. (2020). Lopinavir/ritonavir, ribavirin and IFN-beta combination for nCoV treatment. NCT04276688)[Internet].

[B52] Hung I. F-N. Lung K-C. Tso E. Y-K. Liu R. Chung T. W-H. Chu M-Y. (2020). Triple combination of interferon beta-1b, lopinavir–ritonavir, and ribavirin in the treatment of patients admitted to hospital with COVID-19: An open-label, randomised, phase 2 trial. Lancet 395 (10238), 1695–1704. 10.1016/S0140-6736(20)31042-4 32401715 PMC7211500

[B53] Izzedine H. Launay-Vacher V. Peytavin G. Valantin M. A. Deray G. (2005). Atazanavir: A novel inhibitor of HIV-protease in haemodialysis. Nephrol. Dial. Transplant. 20 (4), 852–853. 10.1093/ndt/gfh684 15772278

[B54] Jonny V. L. Kartasasmita A. S. Amirullah Roesli R. M. Rita C. (2021). Pharmacological treatment options for coronavirus disease-19 in renal patients. Int. J. Nephrol. 2021, 4078713. 10.1155/2021/4078713 34858665 PMC8632427

[B55] Jose S. Nelson M. Phillips A. Phillips Fau - Chadwick D. Chadwick D. Fau - Trevelion R. (2017). Improved kidney function in patients who switch their protease inhibitor from atazanavir or lopinavir to darunavir. AIDS 31 (4), 485–492. 10.1097/QAD.0000000000001353 28121667 PMC5278893

[B56] Kakuda T. N. Brochot A. Tomaka F. L. Vangeneugden T. Van De Casteele T. Hoetelmans R. M. W. (2014). Pharmacokinetics and pharmacodynamics of boosted once-daily darunavir. J. Antimicrob. Chemother. 69 (10), 2591–2605. 10.1093/jac/dku193 24951533

[B57] Kalantari S. Fard S. R. Maleki D. Taher M. T. Yassin Z. Alimohamadi Y. (2021). Comparing the effectiveness of Atazanavir/Ritonavir/Dolutegravir/Hydroxychloroquine and Lopinavir/Ritonavir/Hydroxychloroquine treatment regimens in COVID-19 patients. J. Med. Virology 93 (12), 6557–6565. 10.1002/jmv.27195 34255369 PMC8426706

[B58] Kelton K. Klein T. Murphy D. Belger M. Hille E. McCollam P. L. (2022). Cost-effectiveness of combination of baricitinib and remdesivir in hospitalized patients with COVID-19 in the United States: A modelling study. A Model. Study 39 (1), 562–582. 10.1007/s12325-021-01982-6 PMC8606629 34807369

[B59] Khalili J. S. Zhu H. Mak N. S. A. Yan Y. Zhu Y. (2020). Novel coronavirus treatment with ribavirin: Groundwork for an evaluation concerning COVID‐19. J. Med. virology 92 (7), 740–746. 10.1002/jmv.25798 32227493 PMC7228408

[B60] Khoo S. H. Fitzgerald R. Fletcher T. Ewings S. Jaki T. Lyon R. (2021). Optimal dose and safety of molnupiravir in patients with early SARS-CoV-2: A phase I, open-label, dose-escalating, randomized controlled study. J. Antimicrob. Chemother. 76 (12), 3286–3295. 10.1093/jac/dkab318 34450619 PMC8598307

[B61] Kirby B. Gordi T. Symonds W. T. Kearney B. P. Mathias A. (Editors) (2013). Population pharmacokinetics of sofosbuvir and its major metabolite (GS-331007) in healthy and HCV-infected adult subjects2013 (RIVER ST, HOBOKEN 07030-5774, NJ USA: WILEY-BLACKWELL), 111.

[B62] Kobayashi M. Yoshinaga T Fau - Seki T. Seki T. Fau - Wakasa-Morimoto C. Wakasa-Morimoto C. Fau - Brown K. W. (2011). *In vitro* antiretroviral properties of S/GSK1349572, a next-generation HIV integrase inhibitor. Antimicrob. Agents Chemother. 55 (2), 813–821. 10.1128/AAC.01209-10 21115794 PMC3028777

[B63] Koh Y. Matsumi S. Das D. Amano M. Davis D. A. Li J. (2007). Potent inhibition of HIV-1 replication by novel non-peptidyl small molecule inhibitors of protease dimerization. J. Biol. Chem. 282 (39), 28709–28720. 10.1074/jbc.M703938200 17635930

[B64] Koteff J. Borland J. Fau - Chen S. Chen S. Fau - Song I. Song I. (2013). A phase 1 study to evaluate the effect of dolutegravir on renal function via measurement of iohexol and para-aminohippurate clearance in healthy subjects. Br. J. Clin. Pharmacol. 75 (4), 990–996. 10.1111/j.1365-2125.2012.04440.x 22905856 PMC3612717

[B65] Kreft K. A-O. Spencer C. A. Raghuram A. (2019). Safety and efficacy of dolutegravir in hemodialysis. Int. J. STD AIDS 30 (6), 530–535. 10.1177/0956462418816785 31074360

[B66] Kute V. B. Shah Pr Goplani K. R. Vanikar A. V. Trivedi H. L. (2011). Successful treatment of critically ill chronic kidney disease patient with multi-organ dysfunction associated with H1N1 infection. Indian J. Nephrol. 21 (1), 59–61. 10.4103/0971-4065.78082 21655174 PMC3109787

[B67] Kutzler H. L. Kuzaro H. A. Serrano O. K. Feingold A. Morgan G. Cheema F. (2021). Initial experience of bamlanivimab monotherapy use in solid organ transplant recipients. Transpl. Infect. Dis. 23 (5), e13662. 10.1111/tid.13662 34081820

[B68] Lagging M. Wejstål R. Norkrans G. Karlström O. Aleman S. Weiland O. (2017). Treatment of hepatitis C virus infection: Updated Swedish guidelines 2016. Infect. Dis. 49 (8), 561–575. 10.1080/23744235.2017.1300682 28293974

[B69] Lexi-comp I. (2018). Drug information handbook: A clinically relevant resource for all healthcare professionals. Hudson, OH: Wolters Kluwer.

[B70] Lian N. Xie H. Lin S. Huang J. Zhao J. Lin Q. (2020). Umifenovir treatment is not associated with improved outcomes in patients with coronavirus disease 2019: A retrospective study. Clin. Microbiol. Infect. 26 (7), 917–921. 10.1016/j.cmi.2020.04.026 32344167 PMC7182750

[B71] Liu E. C. Lee J. H. Loo A. Mazur S. Sultan S. Aull M. (2021). Casirivimab-imdevimab (REGN-COV2) for mild to moderate SARS-CoV-2 infection in kidney transplant recipients. Kidney Int. Rep. 6 (11), 2900–2902. 10.1016/j.ekir.2021.08.032 34514186 PMC8418987

[B72] Luke D. R. Wood Nd Fau - Tomaszewski K. E. Tomaszewski Ke Fau - Damle B. Damle B. (2012). Pharmacokinetics of sulfobutylether-β-cyclodextrin (SBECD) in subjects on hemodialysis. Nephrol. Dial. Transpl. 27 (3), 1207–1212. 10.1093/ndt/gfr472 21868395

[B73] Madelain V. Nguyen T. H. T. Olivo A. De Lamballerie X. Guedj J. Taburet A-M. (2016). Ebola virus infection: Review of the pharmacokinetic and pharmacodynamic properties of drugs considered for testing in human efficacy trials. Clin. Pharmacokinet. 55 (8), 907–923. 10.1007/s40262-015-0364-1 26798032 PMC5680399

[B74] Major R. Selvaskandan H. Makkeyah Y. M. Hull K. Kuverji A. Graham-Brown M. (2020). The exclusion of patients with CKD in prospectively registered interventional trials for COVID-19-a rapid review of international Registry data. J. Am. Soc. Nephrol. 31 (10), 2250–2252. 10.1681/ASN.2020060877 32900842 PMC7608999

[B75] Mambelli E. Gasperoni L. Maldini L. Biagetti C. Rigotti A. (2022). Sotrovimab in SARS-COV-2 chronic hemodialysis patients in the omicron era. Is intradialytic administration feasible? Report of 4 cases. J. Nephrol., 1–4. 10.1007/s40620-022-01449-z PMC9469048 36098880

[B76] Marin R-C. Behl T. Negrut N. Bungau S. (2021). Management of antiretroviral therapy with boosted protease inhibitors—darunavir/ritonavir or darunavir/cobicistat. Biomedicines 9 (3), 313. 10.3390/biomedicines9030313 33803812 PMC8003312

[B77] Meng H. Mao J. Ye Q. (2022). Booster vaccination strategy: Necessity, immunization objectives, immunization strategy, and safety. J. Med. Virology 94 (6), 2369–2375. 10.1002/jmv.27590 35028946

[B78] Mentré F. Taburet A-M. Guedj J. Anglaret X. Keïta S. de Lamballerie X. (2015). Dose regimen of favipiravir for Ebola virus disease. Lancet Infect. Dis. 15 (2), 150–151. 10.1016/S1473-3099(14)71047-3 25435054

[B79] Milburn J. Jones R. Levy J. B. (2017). Renal effects of novel antiretroviral drugs. Nephrol. Dial. Transplant. 32 (3), 434–439. 10.1093/ndt/gfw064 27190354 PMC5837523

[B80] Mirijello A. Piscitelli P. de Matthaeis A. Inglese M. D’Errico M. M. Massa V. (2021). Low eGFR is a strong predictor of worse outcome in hospitalized COVID-19 patients. J. Clin. Med. [Internet] 10 (22), 5224. 10.3390/jcm10225224 34830506 PMC8619033

[B81] Mishima E. Anzai N. Miyazaki M. Abe T. (2020). Uric acid elevation by favipiravir, an antiviral drug. Tohoku J. Exp. Med. 251 (2), 87–90. 10.1620/tjem.251.87 32536670

[B82] Morello J. Rodríguez-Novoa S. Jiménez-Nacher I. Soriano V. (2008). Usefulness of monitoring ribavirin plasma concentrations to improve treatment response in patients with chronic hepatitis C. J. Antimicrob. Chemother. 62 (6), 1174–1180. 10.1093/jac/dkn421 18931138

[B83] Mulangu S. Dodd L. E. Davey R. T. Tshiani Mbaya O. Proschan M. Mukadi D. (2019). A randomized, controlled trial of Ebola virus disease therapeutics. N. Engl. J. Med. 381 (24), 2293–2303. 10.1056/NEJMoa1910993 31774950 PMC10680050

[B84] Nasa P. Shrivastava P. Kulkarni A. Vijayan L. Singh A. (2021). Favipiravir induced nephrotoxicity in two patients of COVID-19. J. Assoc. Phys. India 69, 11–12. 34472793

[B85] Nojomi M. Yassin Z. Keyvani H. Makiani M. J. Roham M. Laali A. (2020). Effect of arbidol (umifenovir) on COVID-19: A randomized controlled trial. BMC Infect. Dis. 20 (1), 954. 10.1186/s12879-020-05698-w 33317461 PMC7734453

[B86] Ortiz A. (2021). Chronic kidney disease is a key risk factor for severe COVID-19: A call to action by the ERA-EDTA. Nephrology, dialysis, transplantation: Official publication of the European dialysis and transplant association - European renal association. Nephrol. Dial. Transpl. 36 (1), 87–94. 10.1093/ndt/gfaa314 PMC7771976 33340043

[B87] Packwood R. Galletta G. Tennyson J. (2020). An unusual case report of COVID-19 presenting with meningitis symptoms and shingles. Clin. Pract. Cases Emerg. Med. 4 (3), 316–320. 10.5811/cpcem.2020.4.47557 32926675 PMC7434230

[B88] Painter W. P. Holman W. Bush J. A. Almazedi F. Malik H. Eraut N. C. J. E. (2021). Human safety, tolerability, and pharmacokinetics of molnupiravir, a novel broad-spectrum oral antiviral agent with activity against SARS-CoV-2. Antimicrob. agents Chemother. 65 (5), e02428–e02520. 10.1128/AAC.02428-20 33649113 PMC8092915

[B89] Patel K. Rayner Cr Fau - Giraudon M. Giraudon M. Fau - Kamal M. A. Kamal Ma Fau - Morcos P. N. Morcos Pn Fau - Robson R. (2015). Pharmacokinetics and safety of oseltamivir in patients with end-stage renal disease treated with automated peritoneal dialysis. Br. J. Clin. Pharmacol. 79 (4), 624–635. 10.1111/bcp.12526 25289522 PMC4386947

[B90] Patel S. Wadhwa M. (2021). Therapeutic use of specific tumour necrosis factor inhibitors in inflammatory diseases including COVID-19. Biomed. Pharmacother. = Biomedecine Pharmacother. 140, 111785. 10.1016/j.biopha.2021.111785 PMC8162906 34126316

[B91] Patone M. Mei X. W. Handunnetthi L. Dixon S. Zaccardi F. Shankar-Hari M. (2022). Risks of myocarditis, pericarditis, and cardiac arrhythmias associated with COVID-19 vaccination or SARS-CoV-2 infection. Nat. Med. 28 (2), 410–422. 10.1038/s41591-021-01630-0 34907393 PMC8863574

[B92] Pawlotsky J-M. (2013). Hepatitis C virus: Standard-of-care treatment. Adv. Pharmacol. 67, 169–215. 10.1016/B978-0-12-405880-4.00005-6 23886001

[B93] Pérez-Sáez M. J. Blasco M. Redondo-Pachón D. Ventura-Aguiar P. Bada-Bosch T. Perez-Flores I. (2020). Use of tocilizumab in kidney transplant recipients with COVID-19. Am. J. Transpl. 20 (11), 3182–3190. 10.1111/ajt.16192 PMC7405397 32654422

[B94] Pilkington V. Pepperrell T. Hill A. (2020). A review of the safety of favipiravir–a potential treatment in the COVID-19 pandemic? J. virus Erad. 6 (2), 45–51. 10.1016/S2055-6640(20)30016-9 32405421 PMC7331506

[B95] Preston S. L. Drusano G. L. Glue P. Nash J. Gupta S. K. McNamara P. (1999). Pharmacokinetics and absolute bioavailability of ribavirin in healthy volunteers as determined by stable-isotope methodology. Antimicrob. agents Chemother. 43 (10), 2451–2456. 10.1128/AAC.43.10.2451 10508023 PMC89499

[B96] Pue M. A. Benet L. Z. (1993). Pharmacokinetics of famciclovir in man. Antivir. Chem. Chemother. 4 (6), 47–55. 10.1177/09563202930040s602

[B97] Ramachandran R. Bhosale V. Reddy H. Atam V. Faridi M. M. A. Fatima J. (2022). Phase III, randomized, double-blind, placebo controlled trial of efficacy, safety and tolerability of antiviral drug umifenovir vs standard care of therapy in non-severe COVID-19 patients. Int. J. Infect. Dis. 115, 62–69. 10.1016/j.ijid.2021.11.025 34801738 PMC8603331

[B98] Ren Z. Luo H. Yu Z. Song J. Liang L. Wang L. (2020). A randomized, open-label, controlled clinical trial of azvudine tablets in the treatment of mild and common COVID-19, a pilot study. Adv. Sci. 7 (19), 2001435. 10.1002/advs.202001435 PMC7404576 35403380

[B99] Roberto P. Francesco L. Emanuela C. Giorgia G. Pasquale N. Sara D. (2020). Current treatment of COVID-19 in renal patients: Hope or hype? Intern. Emerg. Med. 15 (8), 1389–1398. 10.1007/s11739-020-02510-0 32986137 PMC7520511

[B100] Rodríguez-TorresSofosbuvir (Gs-M. (2013). Sofosbuvir (GS-7977), a pan-genotype, direct-acting antiviral for hepatitis C virus infection. Expert Rev. anti-infective Ther. 11 (12), 1269–1279. 10.1586/14787210.2013.855126 24215243

[B101] Ruel T. D. Acosta E. P. Liu J. P. Gray K. P. George K. Montañez N. (2022). Pharmacokinetics, safety, tolerability, and antiviral activity of dolutegravir dispersible tablets in infants and children with HIV-1 (IMPAACT P1093): Results of an open-label, phase 1–2 trial. Lancet HIV 9 (5), e332–e40. 35489377 10.1016/S2352-3018(22)00044-3 PMC9313528

[B102] Sathish J. G. Bhatt S. DaSilva J. K. Flynn D. Jenkinson S. A-O. X. Kalgutkar A. S. (2022). Comprehensive nonclinical safety assessment of nirmatrelvir supporting timely development of the SARS-COV-2 antiviral therapeutic, Paxlovid™. Int. J. Toxicol. 41 (4), 276–290. 10.1177/10915818221095489 35603517 PMC9125132

[B103] Shah P. L. Orton C. M. Grinsztejn B. Donaldson G. C. Crabtree Ramírez B. Tonkin J. (2022). Favipiravir in patients hospitalised with COVID-19 (PIONEER trial): A multicentre, open-label, phase 3, randomised controlled trial of early intervention versus standard care. Lancet Respir. Med. 10.1016/S2213-2600(22)00412-X PMC9891737 36528039

[B104] Shiraki K. Sato N. Sakai K. Matsumoto S. Kaszynski R. H. Takemoto M. (2022). Antiviral therapy for COVID-19: Derivation of optimal strategy based on past antiviral and favipiravir experiences. Pharmacol. Ther. 235, 108121. 10.1016/j.pharmthera.2022.108121 35121001 PMC8806403

[B105] Shiraki K. Yasumoto S. Toyama N. Fukuda H. (2021). Amenamevir, a helicase-primase inhibitor, for the optimal treatment of herpes zoster. Viruses 13 (8), 1547. 10.3390/v13081547 34452412 PMC8402822

[B106] Sinclair S. M. Jones J. K. Miller R. K. Greene M. F. Kwo P. Y. Maddrey W. C. (2017). The ribavirin pregnancy Registry: An interim analysis of potential teratogenicity at the mid-point of enrollment. Drug Saf. 40 (12), 1205–1218. 10.1007/s40264-017-0566-6 28689333 PMC7100215

[B107] Singh A. K. Singh A. Singh R. Misra A. (2022). An updated practical guideline on use of molnupiravir and comparison with agents having emergency use authorization for treatment of COVID-19. Diabetes & metabolic syndrome 16 (2), 102396. 10.1016/j.dsx.2022.102396 35051686 PMC8755553

[B108] Singh A. K. Singh A. Singh R. Misra A. (2022). An updated practical guideline on use of molnupiravir and comparison with agents having emergency use authorization for treatment of COVID-19. Clin. Res. Rev. 16 (2), 102396. 10.1016/j.dsx.2022.102396 PMC8755553 35051686

[B109] Singh A. K. Singh A. Singh R. Misra A. (2021). Molnupiravir in COVID-19: A systematic review of literature. Diabetes & Metabolic Syndrome Clin. Res. Rev. 15 (6), 102329. 10.1016/j.dsx.2021.102329 PMC8556684 34742052

[B110] Smolders E. J. de Kanter C. T. M. M. van Hoek B. Arends J. E. Drenth J. P. H. Burger D. M. (2016). Pharmacokinetics, efficacy, and safety of hepatitis C virus drugs in patients with liver and/or renal impairment. Drug Saf. 39 (7), 589–611. 10.1007/s40264-016-0420-2 27098247 PMC4912979

[B111] Soto K. Campos P. Manso R. Antunes A. M. Morello J. Perazella M. A. (2019). Severe acute kidney injury and double tubulopathy due to dual toxicity caused by combination antiretroviral therapy. Kidney Int. Rep. 4 (3), 494–499. 10.1016/j.ekir.2018.11.014 30899878 PMC6409285

[B112] Steiger S. Rossaint J. Zarbock A. Anders H. J. (2022). Secondary immunodeficiency related to kidney disease (SIDKD)-Definition, unmet need, and mechanisms. J. Am. Soc. Nephrol. 33 (2), 259–278. 10.1681/ASN.2021091257 34907031 PMC8819985

[B113] Sulkowski M. Telep L. E. Colombo M. Durand F. Reddy K. R. Lawitz E. (2022). Sofosbuvir and risk of estimated glomerular filtration rate decline or end‐stage renal disease in patients with renal impairment. Alimentary Pharmacol. Ther. 55 (9), 1169–1178. 10.1111/apt.16830 PMC9313579 35235245

[B114] Sun D. (2020). Remdesivir for treatment of COVID-19: Combination of pulmonary and IV administration may offer aditional benefit. AAPS J. 22 (4), 77. 10.1208/s12248-020-00459-8 32458279 PMC7250281

[B115] Taneja S. Duseja A. De A. Mehta M. Ramachandran R. Kumar V. (2018). Low-dose sofosbuvir is safe and effective in treating chronic hepatitis C in patients with severe renal impairment or end-stage renal disease. Dig. Dis. Sci. 63 (5), 1334–1340. 10.1007/s10620-018-4979-6 29484572

[B116] Tarighi P. Eftekhari S. Chizari M. Sabernavaei M. Jafari D. Mirzabeigi P. (2021). A review of potential suggested drugs for coronavirus disease (COVID-19) treatment. Eur. J. Pharmacol. 895, 173890. 10.1016/j.ejphar.2021.173890 33482181 PMC7816644

[B117] Terakawa K. Katagiri D. Shimada K. Sato L. Takano H. (2022). Safety of casirivimab/imdevimab administration in a SARS-CoV-2 positive maintenance dialysis patient in Japan. Cen. Case Rep. 11 (3), 328–332. 10.1007/s13730-021-00671-1 35000134 PMC8742683

[B118] Thakare S. Gandhi C. Modi T. Bose S. Deb S. Saxena N. (2021). Safety of remdesivir in patients with acute kidney injury or CKD. Kidney Int. Rep. 6 (1), 206–210. 10.1016/j.ekir.2020.10.005 33073066 PMC7547837

[B119] Tiec C. L. Barrail A. Goujard C. Taburet A-M. (2005). Clinical pharmacokinetics and summary of efficacy and tolerability of atazanavir. Clin. Pharmacokinet. 44 (10), 1035–1050. 10.2165/00003088-200544100-00003 16176117

[B120] Toots M. Yoon J-J. Cox R. M. Hart M. Sticher Z. M. Makhsous N. (2019). Characterization of orally efficacious influenza drug with high resistance barrier in ferrets and human airway epithelia. Sci. Transl. Med. 11 (515), eaax5866. 10.1126/scitranslmed.aax5866 31645453 PMC6848974

[B121] US Food and Drug Administration (2021). Fact sheet for healthcare providers: Emergency use authorization for paxlovid. Available at: https://www.fda.gov/media/155050/download .(Accessed May 2, 2022).

[B122] Varghese V. Rodriguez R. Self S. Velez J. C. Q. (2020). Atazanavir crystal–induced chronic granulomatous interstitial nephritis. Kidney Int. Rep. 5 (7), 1106–1110. 10.1016/j.ekir.2020.04.007 32647771 PMC7335961

[B123] Vena A. Cenderello G. Balletto E. Mezzogori L. Santagostino Barbone A. Berruti M. (2021). Early administration of bamlanivimab in combination with etesevimab increases the benefits of COVID-19 treatment: Real-World experience from the liguria region. J. Clin. Med. 10 (20), 4682. 10.3390/jcm10204682 34682805 PMC8538905

[B124] Waldenström J. Westin J. Nyström K. Christensen P. Dalgard O. Färkkilä M. (2016). Randomized trial evaluating the impact of ribavirin mono-therapy and double dosing on viral kinetics, ribavirin pharmacokinetics and anemia in hepatitis C virus genotype 1 infection. PLoS One 11 (5), e0155142. 10.1371/journal.pone.0155142 27167219 PMC4864304

[B125] Wang Y. Zhu L-Q. (2020). Pharmaceutical care recommendations for antiviral treatments in children with coronavirus disease 2019. World J. Pediatr. 16 (3), 271–274. 10.1007/s12519-020-00353-5 32166483 PMC7090514

[B126] Warren T. K. Jordan R. Lo M. K. Ray A. S. Mackman R. L. Soloveva V. (2016). Therapeutic efficacy of the small molecule GS-5734 against Ebola virus in rhesus monkeys. Nature 531 (7594), 381–385. 10.1038/nature17180 26934220 PMC5551389

[B127] Wempe M. F. Anderson P. L. (2011). Atazanavir metabolism according to CYP3A5 status: An *in vitro*-*in vivo* assessment. Drug Metabolism Dispos. 39 (3), 522–527. 10.1124/dmd.110.036178 PMC3061561 21148251

[B128] WHO Solidarity Trial Consortium (2022). Remdesivir and three other drugs for hospitalised patients with COVID-19: Final results of the WHO solidarity randomised trial and updated meta-analyses. Lancet 399 (10339), 1941–1953. 10.1016/S0140-6736(22)00519-0 35512728 PMC9060606

[B129] Wiemer A. J. (2020). Metabolic efficacy of phosphate prodrugs and the remdesivir paradigm. ACS Pharmacol. Transl. Sci. 3 (4), 613–626. 10.1021/acsptsci.0c00076 32821882 PMC7409933

[B130] Wyatt C. M. Chaudhari J. Miao S. Krishnasami Z. Hellinger J. Levey A. S. (2020). Ritonavir-boosted protease inhibitors do not significantly affect the performance of creatinine-based estimates of GFR. Kidney Int. Rep. 5 (5), 734–737. 10.1016/j.ekir.2020.01.020 32405595 PMC7210603

[B131] Yoon H. Rhew K. Y. (2013). Famciclovir as an antiviral agent for a patient with acute renal failure. Int. J. Clin. Pharm. 35 (2), 173–175. 10.1007/s11096-012-9737-9 23277418

[B132] Yoon J-J. Toots M. Lee S. Lee M-E. Ludeke B. Luczo J. M. (2018). Orally efficacious broad-spectrum ribonucleoside analog inhibitor of influenza and respiratory syncytial viruses. Antimicrob. agents Chemother. 62 (8), e00766–e00818. 10.1128/AAC.00766-18 29891600 PMC6105843

[B133] Yücel H. E. (2022). A case of acute renal failure with COVID-19 under Molnupiravir treatment. Med. Sci. Discov. 9 (6), 371–374. 10.36472/msd.v9i6.749

[B134] Zein A. F. M. Z. Sulistiyana C. S. Raffaello W. M. Wibowo A. Pranata R. (2022). Sofosbuvir with daclatasvir and the outcomes of patients with COVID-19: A systematic review and meta-analysis with GRADE assessment. Postgrad. Med. J. 98 (1161), 509–514. 10.1136/postgradmedj-2021-140287 37066509

[B135] Zendehdel A. Bidkhori M. Ansari M. Jamalimoghaddamsiyahkali S. Asoodeh A. (2022). Efficacy of oseltamivir in the treatment of patients infected with Covid-19. Ann. Med. Surg. (Lond) 77, 103679. 10.1016/j.amsu.2022.103679 35531426 PMC9054703

[B136] Zhang J-L. Li Y-H. Wang L-L. Liu H-Q. Lu S-Y. Liu Y. (2021). Azvudine is a thymus-homing anti-SARS-CoV-2 drug effective in treating COVID-19 patients. Signal Transduct. Target. Ther. 6 (1), 414. 10.1038/s41392-021-00835-6 34873151 PMC8646019

[B137] Zhang X. W. Yap Y. L. (2004). The 3D structure analysis of SARS-CoV S1 protein reveals a link to influenza virus neuraminidase and implications for drug and antibody discovery. Theochem 681 (1), 137–141. 10.1016/j.theochem.2004.04.065 32287547 PMC7126208

[B138] Zhou X. Hou H. Yang L. Ding G. Wei T. Li C. (2021). Arbidol is associated with increased in-hospital mortality among 109 patients with severe COVID-19: A multicenter, retrospective study. J. Glob. Health 11, 05017. 10.7189/jogh.11.05017 34326998 PMC8284661

